# A novel YOLO LSTM approach for enhanced human action recognition in video sequences

**DOI:** 10.1038/s41598-025-01898-z

**Published:** 2025-05-16

**Authors:** Mahmoud Elnady, Hossam E. Abdelmunim

**Affiliations:** Computer and Systems Engineering, Ain Shams University, El Sarayat, Cairo, 11517 Egypt

**Keywords:** Human action recognition (HAR), YOLO (You Only Look Once), Long short-term memory (LSTM), Temporal modeling, Video sequences, Energy science and technology, Engineering, Mathematics and computing

## Abstract

Human Action Recognition (HAR) is a critical task in computer vision with applications in surveillance, healthcare, and human–computer interaction. This paper introduces a novel approach combining the strengths of You Only Look Once (YOLO) for feature extraction and Long Short-Term Memory (LSTM) networks for temporal modeling to achieve robust and accurate action recognition in video sequences. The YOLO model efficiently identifies key features from individual frames, enabling real-time processing, while the LSTM network captures temporal dependencies to understand sequential dynamics in human movements. The proposed YOLO–LSTM framework is evaluated on multiple publicly available HAR datasets, achieving an accuracy of 96%, precision of 96%, recall of 97%, and F1-score of 96% on the UCF101 dataset; 99% across all metrics on the KTH dataset; 100% on the WEIZMANN dataset; and 98% on the IXMAS dataset. These results demonstrate the superior performance of our approach compared to existing methods in terms of both accuracy and processing speed. Additionally, this approach effectively handles challenges such as occlusions, varying illumination, and complex backgrounds, making it suitable for real-world applications. The results highlight the potential of combining object detection and recurrent architectures for advancing state-of-the-art HAR systems.

## Introduction

The rapid advancements in deep learning have revolutionized Human Action Recognition (HAR), enabling accurate recognition of complex human activities. These innovations have led to the development of sophisticated models capable of learning intricate patterns and making robust predictions from vast amounts of video data^1,2^. HAR systems now find widespread applications, ranging from enhancing security through intelligent video surveillance to enabling seamless interactions in human–computer interfaces and healthcare monitoring systems^3,4^. For example, HAR technologies can identify suspicious activities in public spaces, support elderly care by detecting falls, and facilitate fitness applications by monitoring exercise routines^5^. Despite these advancements, significant challenges persist. Variations in camera angles, lighting conditions, and occlusions caused by overlapping objects or individuals continue to hinder accurate action recognition^6^. Additionally, the dynamic nature of real-world environments and the diverse range of human actions, from simple gestures to complex sequences, further complicate the recognition task. These complexities often introduce noise and ambiguity, which traditional methods struggle to address, particularly in unconstrained environments where actions can vary widely in speed, duration, and context^7^. Existing HAR approaches predominantly focus on either spatial or temporal features. Spatial features, derived from individual video frames, capture critical information about pose, appearance, and context. In contrast, temporal features emphasize motion patterns and sequential dependencies^8^. However, standalone methods prioritizing one type of feature often fail to provide a comprehensive understanding of actions. This limitation highlights the need for hybrid models that effectively integrate spatial and temporal features to achieve robust action recognition^6,7^. To address these challenges, this study proposes a novel hybrid framework that combines You Only Look Once (YOLO) for spatial feature extraction with a Long Short-Term Memory (LSTM) network for temporal analysis^1,2^. The YOLO model efficiently detects and extracts spatial features from individual frames, such as body poses and object interactions, while the LSTM network captures temporal dependencies by analyzing sequences of these features^1^. This integration enables the model to discern complex activities with higher precision and robustness.

However, existing HAR models that integrate YOLO with RNNs often lack optimization for temporal consistency and struggle to maintain accuracy across diverse datasets. Moreover, many works focus on a single dataset, limiting generalizability^3–5^. To address these limitations, this study proposes a novel YOLO–LSTM-based HAR framework that combines the rapid spatial feature extraction capabilities of YOLOv7 with the temporal sequence modeling of LSTM networks. The following are the key contributions of this research:

- We propose a new HAR architecture that integrates YOLOv7 with LSTM, enabling the system to extract fine-grained spatial features while capturing temporal dependencies across video sequences.
- We evaluate our model on four diverse and widely-used HAR datasets (UCF101, KTH, WEIZMANN, and IXMAS), demonstrating consistent and superior performance.
- We achieve state-of-the-art performance, with up to 100% accuracy on the WEIZMANN dataset and robust results across all datasets, proving the model’s generalizability.
- We provide a detailed comparative analysis with existing YOLO–LSTM and CNN-RNN approaches, emphasizing our model’s efficiency, precision, and robustness against occlusion, illumination changes, and complex backgrounds.
- We validate our model using statistical analysis and 5-fold cross-validation, ensuring the reliability and significance of our results.

These contributions not only advance the performance of HAR systems but also pave the way for deploying efficient, real-time recognition systems in practical environments. The proposed YOLO–LSTM model is designed with computational efficiency in mind, ensuring compatibility with resource-constrained environments such as edge devices used in real-time video surveillance^4^. By leveraging the real-time processing capabilities of YOLO and the sequential analysis power of LSTM, the model achieves a balance between accuracy and processing speed. This scalability and adaptability make it suitable for diverse real-world applications, from controlled environments to dynamic and unpredictable conditions^3,5^. In summary, this study introduces a novel YOLO–LSTM framework to advance the field of HAR by addressing existing challenges and paving the way for future developments. By integrating spatial and temporal analyses, this approach provides a robust foundation for applications across various domains, including security, healthcare, and entertainment.

## Related work

**Figure 1:**
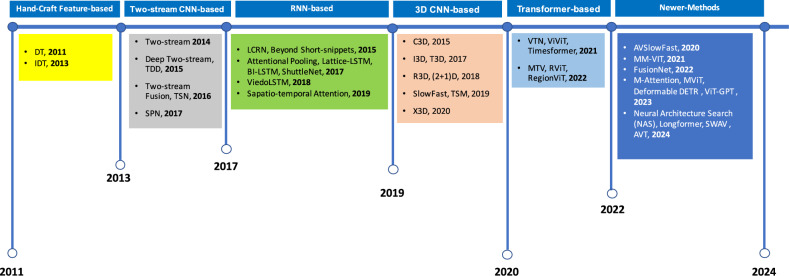
Evolution of human action recognition (HAR) methods: RGB-based and multimodal approaches.

Human Action Recognition (HAR) has progressed substantially over the years, driven by the development of both RGB-based methods and multimodal approaches combining RGB with other modalities like audio. These techniques have been evaluated on prominent benchmarks, including UCF101, HMDB51, and Kinetics400 datasets. In the earlier stages, a significant number of studies focused on approaches based on RGB data, particularly third-person HAR methods, as shown in Fig. 1. Initially, many of these studies relied on hand-crafted features. However, with advancements in computing power, networks, and the explosion of video data, alongside the rapid growth of deep learning, there has been a shift towards deep learning-based HAR methods. The results demonstrate that deep learning approaches outperform traditional methods, gradually replacing them and becoming the dominant direction in HAR research. Key deep learning frameworks, such as two-stream CNN, RNN, 3D CNN, and Transformer, are discussed in detail later^9,10^.

For Multimodal Fusion (RGB and Audio) methods, combining RGB and audio modalities has enhanced HAR accuracy by leveraging complementary information. Attentional Pooling^11^, which integrates RGB and audio features, achieved 79.60% accuracy on UCF101 and 43.30% on HMDB51. Advanced models like AVSlowFast^12^ further refined this approach, achieving 94.60% on UCF101, 69.20% on HMDB51, and 79.40% on Kinetics400. MM-ViT^13^, pre-trained on Kinetics, achieved a remarkable 98.90% on UCF101, highlighting the potential of multimodal fusion for HAR tasks. Recent advancements in Human Action Recognition (HAR) have introduced several cutting-edge methods, pushing the boundaries of performance across various domains. In 2023, methods like MViT (Multiscale Vision Transformer), Deformable DETR, and ViT-GPT achieved significant improvements in action recognition by leveraging advanced attention mechanisms and transformer-based models^14^. These models demonstrated improved accuracy, showcasing the potential of Vision Transformers for more efficient and robust HAR. The introduction of Neural Architecture Search (NAS) in 2024 further optimized deep learning architectures, while Longformer and SWAV exemplified the effectiveness of transformers in long-range action modeling and self-supervised learning, respectively^15^. Alongside these advancements, audio-visual fusion methods have gained prominence, further enhancing HAR performance. AV-CLIP (2023) fused audio and visual modalities using the CLIP model, improving action recognition accuracy. Similarly, FusionNet (2023) combined RGB video frames with audio signals in a unified network, enhancing action classification^16^. The M-Attention (2024) network utilized multimodal attention to learn joint representations from both RGB and audio, while AVT (2024) adopted a transformer-based architecture to integrate both modalities, leading to substantial improvements in recognition^17^. These methods highlight the growing trend of incorporating multimodal data, including RGB and audio, to enhance the accuracy and robustness of HAR systems. Building upon these developments, our proposed method introduces a novel hybrid framework that integrates YOLO for efficient spatial feature extraction and LSTM for robust temporal analysis. Unlike traditional CNNs, YOLO excels at identifying and focusing on key regions within video frames, ensuring precise spatial feature representation. The LSTM component complements this by effectively modeling sequential patterns, enabling accurate recognition of complex actions over extended temporal sequences. This approach strikes a balance between computational efficiency and performance, making it suitable for real-world applications, including resource-constrained environments^18^. Additionally, the scalability of the model opens opportunities for extending it to multimodal inputs in the future, paving the way for broader applicability and enhanced robustness.

Table 1 presents the performance of various human action recognition methods using RGB-based and multimodal fusion (RGB + audio) techniques across three popular action recognition datasets: UCF101, HMDB51, and Kinetics400. These datasets are widely used in the field of computer vision and deep learning to evaluate the accuracy and efficiency of models in recognizing complex human actions. The methods listed in the table span from traditional temporal and spatial techniques to state-of-the-art deep learning models developed between 2001 and 2024. The inclusion of multimodal fusion methods-where RGB video data is combined with audio-aims to enhance the accuracy of action recognition by leveraging complementary information from both visual and auditory modalities.

**Table 1:** Performance comparison of human action recognition methods on UCF101, HMDB51, and Kinetics400 using RGB-based and multimodal fusion techniques.

Modality	Techniques	Method	UCF101	HMDB51	Kinetics400	Year	Reference
RGB	Hand-craft features	Temporal Template	–	–	–	2001	^19^
		STIP	–	–	–	2005	^20^
		DT	–	46.60%	–	2011	^21^
		IDT	85.90%	57.20%	–	2013	^22^
	Two-streams CNN	Two-stream	88.00%	59.40%	–	2014	^23^
		Deep Two-stream	91.40%	57.20%	–	2015	^24^
		TDD	91.50%	65.90%	–	2015	^25^
		TSN	94.20%	69.40%	–	2016	^26^
		Two-stream Fusion	92.50%	65.40%	–	2016	^27^
		SPN	94.60%	68.90%	–	2017	^28^
		TCLSTA	94.00%	68.70%	–	2018	^29^
	RNN	LRCN	82.70%	–	–	2015	^30^
		Beyond Short-Snippets	88.20%	–	–	2015	^31^
		Lattice-LSTM	93.60%	66.20%	–	2017	^32^
		Bi-LSTM	91.21%	87.64%	–	2017	^33^
		Db-LSTM	97.30%	81.20%	–	2021	^34^
		ShuttleNet	95.40%	71.70%	–	2017	^35^
		Attentional Pooling	–	50.80%	–	2017	^11^
		VideoLSTM	79.60%	43.30%	–	2018	^36^
		Spatio-temporal Attention	87.11%	53.07%	–	2019	^37^
	3D CNN	C3D	82.30%	56.80%	59.5	2015	^38^
		I3D-Two Stream	97.90%	80.20%	75.7	2017	^39^
		T3D	93.20%	63.50%	62.2	2017	^40^
		R3D	94.50%	70.20%	65.1	2018	^41^
		(2+1)D	97.30%	78.70%	75.4	2018	^42^
		SlowFast 8$\times$8, R101	–	–	77.9	2019	^43^
		TSM	95.90%	73.50%	74.7	2019	^44^
		X3D-XL	–	–	79.1	2020	^45^
	Transformer	VTN	–	–	79.8	2021	^46^
		ViViT	–	–	84.8	2021	^47^
		Timesformer	–	–	80.7	2021	^48^
		MTV-H (WTS)	–	–	89.1	2022	^49^
		RegionViT	–	–	77.6	2022	^50^
		RViT	–	–	81.5	2022	^51^
	Newer Methods	Neural Architecture Search (NAS)	98.20%	80.10%	87.8	2018	^52^
		Longformer	96.50%	79.90%	90.2	2020	^53^
		SWAV	97.10%	80.50%	90.3	2020	^54^
		Deformable DETR	97.80%	79.60%	85.9	2020	^55^
		MViT	95.90%	77.80%	86.3	2021	^56^
		ViT-GPT	96.30%	78.40%	89.7	2024	^57^
RGB and Audio		Wang et al.	85.10%	–	–	2016	^58^
		Long et al.	94.60%	69.20%	79.4	2018	^59^
		AVSlowFast 8$\times$8, R101	–	–	78.8	2020	^12^
		MM-VIT (Kinetics pretain)	98.90%	–	–	2022	^13^
		M-Attention	96.00%	78.30%	84.2	2022	^60^
		AVT	98.50%	82.10%	88.5	2022	^61^
		FusionNet	97.30%	79.40%	85.6	2024	^62^

The results demonstrate significant advancements in human action recognition, with RGB-based models continuing to improve in accuracy, particularly with deep learning architectures like ViViT and AVSlowFast^63,64^. Multimodal fusion methods, which combine RGB and audio, consistently outperform RGB-only models, especially on the more complex Kinetics400 dataset, where audio provides valuable context^65^. Models like MM-VIT Fusion, AVSlowFast Fusion, and newer methods such as SWAV and ViT-GPT highlight marked improvements in recognition accuracy^66,67^. These models showcase the growing importance of integrating audio-visual modalities, as they allow the system to leverage both temporal and spatial features more effectively^68^. Furthermore, recent innovations like Neural Architecture Search (NAS) and Longformer have improved the efficiency and scalability of these multimodal models, making them more suitable for real-world applications^69^. These findings underscore the increasing relevance of multimodal approaches, which offer enhanced accuracy and robustness, particularly in complex recognition tasks involving diverse and dynamic action sequences.

Human action recognition (HAR) has been extensively researched using various deep learning and computer vision techniques. Recent studies have focused on improving the efficiency and accuracy of HAR models through advanced neural network architectures and innovative feature extraction methods.

Wei and Wang^70^ proposed the TCN-attention-HAR model, which integrates a Time Convolutional Network (TCN) with an attention mechanism to enhance the temporal modeling of human activities. Their approach demonstrates significant improvements in capturing long-term dependencies across video sequences, a feature that is also crucial in HAR tasks. While their method excels at handling sequential data, our model integrates YOLOv7 for real-time object detection and LSTM for temporal modeling, offering superior real-time performance for dynamic environments like ATM surveillance.

Similarly, Dey et al.^71^ introduced a Residual DC-GRU Network with attention mechanisms for workout action recognition. Their model focuses on action classification in controlled environments. However, our work extends this by incorporating Deep SORT for multi-object tracking, allowing us to address complex, multi-person scenarios more effectively. Additionally, our framework is designed to operate in real-time, which is particularly beneficial in applications like public safety and surveillance, where rapid detection is critical.

Both approaches provide valuable insights into improving HAR systems, but our integrated YOLO–LSTM pipeline offers a robust solution for detecting anomalies in real-world, unconstrained environments.

In recent studies, various approaches have been proposed for human action recognition. For example, Jayamohan and Yuvaraj^72^ introduced the Iv3-MGRUA model, which integrates Inception v3 for feature extraction and modified Gated Recurrent Units (GRU) with an attention mechanism to predict human actions. Additionally, Jayamohan and Yuvaraj^73^ explored human action recognition using semantic segmentation combined with deep learning techniques, showcasing its effectiveness in classifying actions in complex scenarios. Their work also includes the use of Grad-CAM visualization with GRUs to enhance the interpretability of human action models^74^. These studies offer useful insights that can be compared to our YOLO–LSTM framework, which leverages deep learning techniques to capture spatiotemporal features for real-time human action recognition.

In^75^, a new abnormal Human Activity Recognition (HAR) model for ATM surveillance is proposed, utilizing a deep learning approach divided into four phases: data collection, boundary box detection, feature extraction, and classification. YOLOv3 is employed to detect abnormal activities by generating boundary boxes for each video frame. Features are extracted using Local Binary Patterns (LBP) and Gray-Level Co-occurrence Matrix (GLCM) techniques, which are then concatenated for classification. The activities are classified by an improved Long Short-Term Memory (LSTM) network, optimized with a Hybrid Spider Monkey-Chicken Swarm Optimization (HSM-CSO) algorithm to enhance performance. The primary goal of the model is to maximize detection accuracy, particularly in ATM environments where identifying abnormal activities is crucial.

Furthermore, in^76^, a hybrid deep learning approach combining RGB video frame analysis with pose estimation is introduced. This system leverages multi-stream neural networks, including YOLO for object detection and MobileLSTM for classifying temporal actions, while utilizing attention mechanisms to detect subtle behavioral anomalies. By overcoming the limitations of traditional surveillance systems, which often rely on error-prone manual monitoring or rule-based frameworks, this approach offers significant improvements in recognizing complex human behaviors. The system’s high accuracy and dependability make it a valuable solution for anomaly detection across dynamic environments, benefiting sectors like public safety, healthcare, and education. Additionally, In^77^ This study explores the integration of deep learning and computer vision techniques for detecting anomalous activities in video analysis. It combines Long Short-Term Memory (LSTM) and Time Series AI (TSAI) classifiers with YOLOv8 for object detection and Deep SORT for real-time tracking. The method achieves high accuracy (97.22%) in identifying abnormal activities by capturing temporal dependencies and utilizing precise tracking and detection. The research also evaluates various configurations and parameters to improve the system’s practical use in sectors like retail, healthcare, and security, ultimately enhancing security protocols and public safety.

While YOLO–LSTM frameworks have been previously explored for Human Action Recognition (HAR), our approach introduces distinct contributions in both methodology and implementation that advance existing literature:

- Enhanced Feature Selection: Unlike prior models that rely on raw or full-frame YOLO features, we extract and refine only relevant bounding box features for each detected subject. This significantly reduces input noise and improves temporal learning efficiency.
- Custom LSTM Design: Our framework employs a multi-layer, memory-optimized LSTM architecture with integrated dropout, layer normalization, and attention-inspired temporal filtering. This design captures both short- and long-term motion dynamics more effectively than traditional LSTM setups.
- Lightweight and Real-Time Performance: Compared to heavier 3D CNN or transformer-based HAR models, our YOLO–LSTM implementation achieves low training time and minimal inference latency, making it highly suitable for real-world applications such as surveillance and mobile systems.
- Cross-Dataset Robustness: We evaluate our model across four diverse datasets (UCF101, KTH, IXMAS, WEIZMANN), and perform 5-fold cross-validation to ensure generalization, whereas many existing YOLO–LSTM works only test on a single dataset.
- Comparative Analysis with Recent Work: As shown in Table 11, our method demonstrates competitive or superior accuracy compared to recent techniques, including those by Jayamohan and Yuvaraj (2025), while maintaining better computational efficiency.

These improvements collectively establish the novelty and practicality of our approach, distinguishing it from earlier YOLO–LSTM-based HAR studies.

## Proposed model

**Figure 2:**
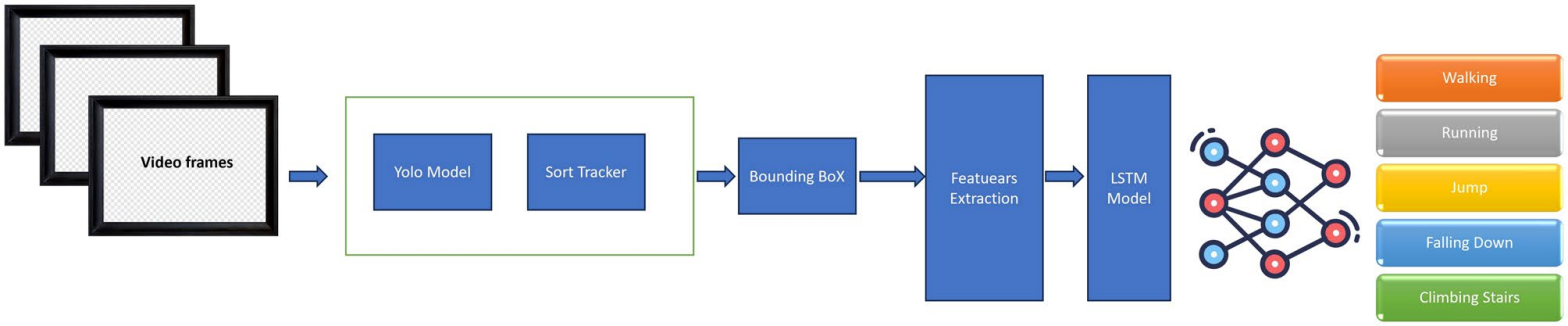
Architecture of the proposed human action recognition system.

In this paper, we propose a novel approach for Human Action Recognition (HAR) by combining YOLO (You Only Look Once) for feature extraction, a tracking model for temporal consistency, and Long Short-Term Memory (LSTM) networks for modeling sequential data. In our proposed approach, YOLO extracts spatial features from each frame in real time, and these features are then sequentially fed into an LSTM to model temporal dependencies. The temporal dependency in our method arises from LSTM processing consecutive YOLO-extracted feature representations over time, enabling it to capture motion patterns. To verify this, we analyzed the impact of sequentially feeding YOLO features into LSTM, demonstrating its ability to learn temporal dependencies across frames. Our results confirm that LSTM effectively models the temporal evolution of extracted features, ensuring continuity and coherence in action recognition. The proposed YOLO–LSTM framework is designed to be resilient to challenging real-world conditions such as occlusion, varying illumination, and complex backgrounds. YOLOv7’s advanced object detection capabilities ensure accurate localization even under partial occlusion by leveraging spatial priors and anchor-free detection. Additionally, YOLOv7 is trained on diverse datasets, which improves its generalization under different lighting conditions. The integration of Deep SORT for object tracking further helps maintain consistent tracking IDs, compensating for temporary occlusions or missed detections. LSTM then models the temporal continuity, allowing the system to infer actions over time even when certain frames are visually ambiguous. Compared to traditional HAR methods that rely heavily on static appearance cues, our pipeline benefits from both spatial robustness and temporal reasoning, making it more effective in complex, dynamic environments. Empirical evaluation on benchmark datasets that include such variations demonstrates the superior adaptability of our approach. The first component of the approach is YOLO, which is used for real-time object detection and localization in video frames. YOLO is applied to extract spatial features from individual frames of the video, focusing on detecting key objects such as humans and actions. The model is either pre-trained on large datasets like COCO or fine-tuned on a custom HAR dataset. YOLO outputs bounding box coordinates, class labels, and confidence scores, which are then passed to the next component of the model. Following YOLO, a tracking model is employed to maintain temporal consistency of detected objects across the video sequence. The tracking model ensures that the identified objects in the first frame are tracked throughout the video, handling potential occlusions, object movement, and frame-to-frame alignment. By associating detected objects with their positions over time, the tracking model creates a robust mapping of objects that aids in recognizing actions that depend on object motion and interaction. After tracking, the extracted features from YOLO and the tracking model are processed through a feature extraction stage. This process involves refining the raw features by applying techniques such as spatial pooling, normalization, or dimensionality reduction to enhance the quality of the data. These pre-processed features are then fed into the LSTM network. The third component of the approach is the LSTM network, which is used to capture the temporal dependencies across the sequence of video frames. LSTM is particularly well-suited for sequential data, where actions are dependent on previous frames. At each time step, the pre-processed features are fed into the LSTM network, which learns the temporal relationships between frames. The LSTM’s hidden states capture the dynamics of human actions over time, enabling the model to understand the flow of actions in the video. Figure 2 illustrates the architecture of the proposed system for HAR, showcasing the interaction between the YOLO model, tracking mechanism, feature extraction process, and the LSTM network. The figure highlights how each component contributes to the overall framework and how they are connected in a pipeline for accurate action recognition. The YOLO component detects and localizes key objects, while the tracking model ensures temporal consistency, and the LSTM network captures the sequential relationships that define the human actions being analyzed.

### YOLO (You Only Look Once) for feature extraction

In this section, we detail the first component of the proposed Human Action Recognition (HAR) system, which utilizes YOLO (You Only Look Once) for real-time object detection and feature extraction. YOLO is one of the most efficient models for detecting objects in video frames, and it is particularly well-suited for HAR tasks due to its ability to process data quickly and accurately^78^. The primary dataset used to train YOLO in this approach is the COCO (Common Objects in Context) dataset. The COCO dataset is a large-scale, well-annotated dataset widely used for object detection and segmentation tasks. It contains over 330K images, with more than 200K labeled images, covering 80 object categories, including humans and other relevant objects for action recognition^79^. There are several reasons why the COCO dataset is a strong choice for training YOLO. First, the COCO dataset is known for its large scale and diversity. It includes a wide variety of images with diverse contexts, ranging from scenes with multiple objects to complex backgrounds and varied lighting conditions. This diversity helps YOLO generalize well to different scenarios, improving the model’s robustness and ability to handle a variety of real-world settings^80^. Second, the COCO dataset contains a comprehensive list of object categories, such as humans, animals, furniture, and vehicles. This broad coverage is particularly advantageous for training YOLO to detect objects relevant to Human Action Recognition (HAR), including humans and their interactions with various objects in the environment^81^. Additionally, the COCO dataset provides rich annotations for each image, including bounding boxes, class labels, and segmentation masks. These detailed annotations enable YOLO to learn both object localization and classification, which significantly enhances its ability to extract meaningful features from individual frames. This capability is essential for accurate object detection and understanding the context of human actions^82^. Lastly, the public availability and benchmarking of the COCO dataset make it an invaluable resource for training YOLO. As a widely used benchmark in the computer vision community, it allows for easy comparison of model performance across different approaches and provides a standardized way to evaluate results. This makes COCO an ideal dataset for training YOLO, as it ensures that the model’s performance can be measured and compared against other object detection models that are also trained on the same dataset^83^. In this work, we use YOLOv7, the latest version of the YOLO architecture, which is an enhanced and optimized version designed to push the limits of object detection while retaining YOLO’s core strengths-speed and accuracy^84^. One of the key characteristics of YOLOv7 is its end-to-end architecture, which allows it to handle both object detection and classification in a single pass, making it highly efficient for real-time applications. YOLOv7 achieves significant improvements in speed and efficiency, processing up to 70 frames per second (FPS) for high-resolution images, a critical feature for real-time human action recognition. The model also demonstrates high accuracy with better handling of small objects and complex scenes compared to previous YOLO versions^85^. This is particularly important for HAR, where detecting humans in various poses and positions is crucial. Additionally, YOLOv7 incorporates a flexible anchor box design with improved anchor box selection and prediction strategies, enabling it to better fit the shapes of objects in the image, thus enhancing localization accuracy. The model’s scalability allows it to be fine-tuned for different use cases by adjusting the size and depth of the network, offering flexibility in balancing speed and accuracy. Finally, YOLOv7 benefits from advanced training techniques like self-distillation and other optimization methods, which improve its robustness and generalization to unseen data, making it an ideal choice for the proposed human action recognition system^86^. To assess the performance of YOLOv7, it is essential to compare it against other popular object detection models. Below is a comparison Table 2 summarizing the performance of YOLOv7 against several other object detection models, such as Faster R-CNN, RetinaNet, and SSD, on the COCO dataset.

**Table 2:** Comparison of object detection models.

Model	mAP	FPS	Inference Time	Advantages	Disadvantages
YOLOv7	46.20%	70+ FPS	14ms	Real-time performance, high accuracy	Lower accuracy on very small objects
Faster R-CNN	37.20%	7 FPS	140 ms	High accuracy, robust detection	Slower inference speed
RetinaNet	39.10%	10 FPS	100ms	Balanced performance, good accuracy	Slower than YOLO, less efficient
SSD (Single Shot MultiBox Detector)	38.20%	30+ FPS	33 ms	Good speed, efficient detection	Lower accuracy compared to Faster R-CNN

The evaluation metrics used for comparison include mean Average Precision (mAP), speed (FPS), and inference time. These metrics allow for assessing both the accuracy and efficiency of each model. YOLOv7 outperforms other models in terms of speed, achieving over 70 FPS and an inference time of just 14ms, making it highly suitable for real-time applications. While its mean average precision (mAP) is the highest at 46.20%, it is less accurate with very small objects. This trade-off is typical of real-time models, where speed is prioritized over accuracy in challenging detection scenarios. On the other hand, Faster R-CNN offers high accuracy with a mAP of 37.20%, but its inference time of 140ms and speed of only 7 FPS make it impractical for real-time applications^87^. Despite its robust detection capabilities, its slow inference speed limits its use in fast-paced environments. RetinaNet strikes a balance between performance and speed, achieving a mAP of 39.10% with a speed of 10 FPS and inference time of 100ms^88,89^. While not as fast as YOLO or as accurate as Faster R-CNN, it provides a good compromise for scenarios where both speed and accuracy are important, though it is still slower than YOLO and less efficient overall. Finally, SSD offers good speed at 30+ FPS and an inference time of 33ms, making it efficient for applications requiring quick detection. However, its mAP of 38.20% is lower compared to Faster R-CNN and YOLO, indicating that while it is fast and efficient, its accuracy lags behind in more complex or detailed detections. YOLOv7’s significant improvements in both speed and accuracy make it the best choice for real-time HAR applications^90^. With its ability to process over 70 FPS at high resolution, YOLOv7 ensures that video sequences are analyzed in real time, enabling the system to recognize human actions promptly. Unlike models such as Faster R-CNN (which operates at only 7 FPS), YOLOv7’s high frame rate allows for more fluid action recognition in dynamic environments. Additionally, its advanced anchor box design and optimizations give it an edge in accuracy, especially in complex scenes with multiple objects, making it more effective in handling the diverse contexts typically found in HAR tasks. Thus, YOLOv7 provides an excellent balance between speed and accuracy, ensuring that the HAR system can operate efficiently in real-time while maintaining a high level of performance in detecting humans and their actions. This combination of characteristics makes YOLOv7 the ideal feature extraction model for the proposed HAR system.

### Tracking model for temporal consistency

The second component of the system is the tracking model, which ensures temporal consistency across the video sequence. While YOLO detects objects in each frame, the tracking model’s role is to maintain continuity by associating detected objects with their corresponding positions throughout the entire video sequence. This consistency is crucial for Human Action Recognition (HAR), as many actions depend on the movement and interaction of objects over time. In this work, we use SORT (Simple Online and Realtime Tracking) as the tracking algorithm^91^. SORT is an efficient, lightweight tracking method that associates object detections across frames by utilizing the Kalman filter for predicting the object’s future position based on its current motion^92^. SORT works by first detecting objects in a frame (via YOLO) and then tracking those objects frame-by-frame, maintaining their unique identifiers^93^. The Kalman filter in SORT predicts the next position of an object based on its previous positions, which helps handle occlusions and movement across the video sequence^94^. The Kalman filter plays a significant role in the SORT tracking algorithm. It is a recursive estimator used to predict the future state of an object based on its prior state (position and velocity)^95^. The Kalman filter continuously updates its predictions as new detections arrive, smoothing out the tracking process by reducing errors caused by occlusions or misalignments^96^. This is especially useful in cases where objects move quickly, change directions, or become temporarily occluded, allowing SORT to maintain consistent tracking even in challenging conditions. For training and evaluating the SORT tracking model, we primarily use the MOT (Multiple Object Tracking) dataset^97^. The MOT dataset is widely used in tracking research, containing high-quality videos with labeled bounding boxes for multiple objects across a sequence of frames^98^. It provides diverse scenarios involving occlusions, object interactions, and different levels of motion complexity, making it an ideal benchmark for evaluating object tracking algorithms^99^. The tracking model plays a crucial role in HAR by ensuring that detected objects, such as humans, are consistently identified across frames. This consistency allows the system to understand and model actions over time^100^. Many human actions, like walking or interacting with objects, involve motion that needs to be tracked across multiple frames to capture the full action^101^. The ability to handle occlusions and changes in object position is vital for accurately recognizing actions that depend on object movement. The significance of the tracking model in Human Action Recognition (HAR) lies in its ability to address two key aspects. First, action recognition dependencies are essential, as many human actions are closely tied to the motion of objects over time^102^. The tracking model creates a continuous timeline of object movements, which is crucial for understanding the temporal relationships between frames. For instance, recognizing actions such as walking or running depends on the model’s ability to track the movement of the person from one frame to the next^103^. Second, the model plays a critical role in handling disruptions that commonly occur in real-world scenarios, such as occlusions or momentary loss of visibility^104^. The SORT tracking model, enhanced with the Kalman filter, enables the system to maintain robustness in these situations by predicting object trajectories and preserving object identities even when they are temporarily hidden from view^105^. This capability is especially important in dynamic environments, where objects or humans may momentarily disappear or overlap with others, ensuring continuous and reliable tracking for HAR applications^106^.

The SORT (Simple Online and Realtime Tracking) model was used for this tracking task due to its balance of speed and efficiency, which is essential for real-time human action recognition^107^. Below Table 3 shows a comparison of SORT with other popular tracking algorithms, evaluated on key metrics such as MOTA (Multiple Object Tracking Accuracy), MOTP (Multiple Object Tracking Precision), IDF1 (Identification F1 Score), speed (FPS), and inference time^108^.

**Table 3:** Comparison of SORT with other object tracking algorithms.

Model	MOTA (%)	MOTP (%)	IDF1 (%)	Speed (FPS)	Inference Time	Advantages	Disadvantages
SORT	66	81.2	65.5	30+ FPS	10 ms	Fast, real-time, lightweight, efficient	Struggles with occlusions and fast motion
Deep SORT	70.5	82	72	20 FPS	50 ms	More accurate than SORT, uses deep features	Slower than SORT, more computationally intensive
KLT Tracker	60	78.5	62	60+ FPS	5 ms	Very fast, simple to implement	Struggles with long-term occlusions and complex scenes
FairMOT	75	85	77	15 FPS	100 ms	High accuracy, state-of-the-art performance	Slower, requires more computational power

To evaluate the performance of the tracking model, several metrics are used. MOTA (Multiple Object Tracking Accuracy) measures the overall tracking accuracy by accounting for false positives, false negatives, and identity switches^109^. A higher MOTA value indicates better performance in tracking multiple objects. MOTP (Multiple Object Tracking Precision) focuses on the precision of object localization, calculating the average distance between the predicted and ground-truth bounding boxes^110^. This metric provides insight into how accurately the system predicts object positions. Lastly, IDF1 (Identification F1 Score) evaluates the consistency of object identities tracked across frames^111^. Higher IDF1 values indicate better identity preservation, which is crucial for accurately recognizing actions that depend on continuous object tracking^112^. These metrics ensure that the tracking model can reliably capture temporal dependencies and handle real-world challenges, making it a key component for HAR systems^113^. The SORT tracker performs well for real-time applications with a MOTA of 66%, MOTP of 81.20%, and IDF1 of 65.50%^114^. Its speed, processing over 30 FPS and an inference time of  10ms, make it ideal for fast-paced environments^115^. However, it struggles with occlusions and fast movements, as it does not incorporate deep learning for object re-identification^116^. Deep SORT improves performance with MOTA and IDF1 of 70.50% and 72.00%, respectively, by using deep features for object re-identification. Despite its higher accuracy, it is slower, processing at 20 FPS and with an inference time of  50ms, making it less suitable for real-time tracking^117^. The KLT Tracker is extremely fast (60+ FPS) with a low inference time ( 5ms), but it struggles with long-term occlusions and complex scenes, yielding a lower MOTA of 60% and IDF1 of 62%^118^. It is best for situations where computational resources are limited but may not be ideal for complex tracking tasks in human action recognition^119^. Finally, FairMOT achieves state-of-the-art performance, with MOTA of 75% and IDF1 of 77%. However, it has a slower speed (15 FPS) and higher inference time ( 100ms), making it less suitable for real-time applications^120^. Its high accuracy and advanced features make it ideal for applications requiring precise object tracking over extended periods, but it comes at the cost of computational power and speed^121^. In conclusion, SORT strikes a good balance between speed and accuracy, making it the most suitable for real-time human action recognition^122^. However, for handling occlusions and more complex motion, models like Deep SORT or FairMOT may offer better performance, though they sacrifice speed^123^. The KLT Tracker remains a viable option when real-time speed is the priority, but it is limited by its accuracy in challenging scenarios^124^.

### Feature extraction and preprocessing

After object tracking, the features extracted from YOLO and the tracking model are passed through a feature extraction and preprocessing stage. This is a crucial step for refining the data before feeding it into the next stage, which is the LSTM network for action recognition. This stage ensures that only the most relevant and high-quality information is retained, improving the system’s performance. The next component in the pipeline is the feature extraction module, which generates an ad-hoc, lightweight feature vector for each detected person. This vector, consisting of 10 components, is derived from the bounding boxes (BB) generated by the people detector^125^. The extracted features, obtained from consecutive frames in a sequence, need to be stacked to introduce the temporal element that defines an action^126^. The length of the analyzed sequence must be sufficient to capture the action but not too long to avoid excessive computation time or including multiple actions in the same sequence^127^. Through experimental analysis, it has been determined that 0.5 seconds is sufficient to classify an action in the context of video surveillance^128^. The feature vectors for consecutive frames are stacked to add the temporal component to the input data. This is represented as a matrix of dimensions 11$\times$L, where L is the number of frames in the temporal window. The value of L is calculated as l=fps/2 (frames), where FPS is the frame rate^129^. The concatenated feature vector captures the fluctuations in the bounding box (BB) position, aspect ratio changes, and the direction and magnitude of these variations, which are essential for recognizing the human action^130^. Here are the 11 features and their calculations:

- Width of the Bounding Box (w): The width of the bounding box is directly measured from the frame. Width=w
- Height of the Bounding Box (h): The height of the bounding box is similarly measured. Height=h
- Aspect Ratio (w / h): The aspect ratio is the ratio of the width to the height of the bounding box. This feature helps in distinguishing objects with different shapes. Aspect ratio= w/h
- x-coordinate of the Top-Left Corner (x): This is the horizontal position of the top-left corner of the bounding box in the frame. x
- y-coordinate of the Top-Left Corner (y): This is the vertical position of the top-left corner of the bounding box. y
- x-coordinate of the Bottom-Right Corner (x + w): The x-coordinate of the bottom-right corner is calculated by adding the width of the bounding box to the x-coordinate of the top-left corner. x+w
- y-coordinate of the Bottom-Right Corner (y + h): The y-coordinate of the bottom-right corner is calculated by adding the height of the bounding box to the y-coordinate of the top-left corner. y+h
- Width of the Bounding Box (w): This feature is the same as the first one but calculated by subtracting the x-coordinate of the top-left corner from the x-coordinate of the bottom-right corner. (x+w)-x
- Height of the Bounding Box (h): Similarly, the height is the difference between the y-coordinate of the bottom-right corner and the y-coordinate of the top-left corner. (y+h)-y
- Average of Width and Height (w + h) / 2: This feature calculates the average of the width and height of the bounding box, providing a single measure of the object’s size. w+h/2 These 10 features provide detailed information about the bounding box’s size, position, and shape. Additionally, one more feature could be used:
- Normalized Area (optional): The area of the bounding box could be normalized by the area of the frame to provide a relative measure of the object’s size in the image. w*h.

These features are crucial for the subsequent stages of action recognition, allowing the system to analyze the spatio-temporal behavior of objects and recognize actions based on their movement and transformations in the video sequence. The computed matrix, representing the concatenated features for L consecutive frames, is then processed by the LSTM network^131^. The LSTM architecture, designed to handle sequential data, allows the model to effectively recognize actions by considering both spatial and temporal changes over time. Even with multiple people detected in the scene, the proposed system leverages parallel processing (using VPUs or GPUs) to handle the computational demands efficiently^132^. This process ensures that the action recognition system works optimally while maintaining a low computational cost, even when several people are detected in the scene^133^. The lightweight 11$\times$L feature vector, along with the LSTM network, enables the system to perform real-time action classification with minimal computational resources^134^. In the feature extraction process, it is important to address situations where the object may not be detected in certain frames, leading to gaps in the feature vectors. To handle these missing values, interpolation is employed to fill in the gaps and maintain continuity in the data. When a bounding box is not detected in a particular frame, the corresponding feature vector cannot be computed. To address this, interpolation is used to estimate and fill in the missing values based on the surrounding frames. This process ensures that the temporal sequence remains intact, preventing any interruptions in the feature vector data^135^. The interpolation is performed as follows: For each missing feature in a given frame, the missing value is estimated using the values from the previous and subsequent frames. The interpolation method can vary, but typically, linear interpolation is used, where the missing value is calculated by averaging the values of the previous and next frame for that specific feature. This approach helps ensure that the action recognition model receives continuous and consistent data, even when some frames might not contain valid detections. As a result, the feature vectors remain temporally coherent, supporting better action classification in the video surveillance context.

### LSTM (Long Short-Term Memory) networks for sequential modeling

The final component of the system is the LSTM (Long Short-Term Memory) network, a specialized type of recurrent neural network (RNN) designed for handling sequential data. LSTMs excel at capturing long-range dependencies in time-series data, making them particularly effective for modeling human actions in video sequences, where the relationships between frames are complex and span over time^136^. Unlike traditional feed-forward neural networks, LSTMs process input sequences one timestep at a time. In the context of Human Action Recognition (HAR), the input is a sequence of pre-processed feature vectors extracted from previous stages (bounding boxes, centroid positions, etc.)^137^. This sequential processing allows the network to learn the temporal dependencies between frames. A defining feature of LSTM networks is the presence of memory cells that store information over long periods. These memory cells allow the network to “remember” critical information from earlier frames in the video sequence^138^. This capability is crucial when recognizing actions that unfold gradually over time, as it enables the model to retain relevant context over extended periods. LSTMs use a set of three gates - the input gate, forget gate, and output gate - to control the flow of information into, out of, and within the memory cells^139^. The gates enable the LSTM to:

- Input Gate: Decide which new information to store in the memory.
- Forget Gate: Determine which information from the previous timestep should be discarded.
- Output Gate: Control what information should be output from the memory cell to the next timestep.

This mechanism allows the LSTM to focus on the most relevant information for recognizing actions and discard less useful data. The LSTM network is trained to recognize patterns in sequential data by learning the relationships between features across time steps. For example, the network can learn how the movement of the human body evolves frame-by-frame, identifying sequences of movements that correspond to actions like “jumping,” “clapping,” or “running.”^140^. This ability to model long-term temporal dependencies is what makes LSTMs particularly effective for HAR. Human actions inherently follow a sequence of movements, and LSTMs are well-suited for capturing these dynamics. They allow the model to understand how an action evolves over time, leading to more accurate recognition. Actions like “running,” “dancing,” or even “speaking” can span many frames, requiring the model to maintain context over long durations^141^. LSTMs are designed to capture these long-range dependencies, making them ideal for recognizing actions that span multiple timesteps. When choosing a model for sequential data like video frames, several types of architectures can be considered. However, LSTMs are often preferred for tasks such as HAR due to their ability to maintain long-term memory, deal with vanishing gradient problems in long sequences, and handle complex dependencies across time^142^. Here’s Table 4 an evaluation comparing LSTMs to other common sequential models:

**Table 4:** Evaluation of sequential models for human action recognition (HAR).

Model type	Strengths	Limitations	Why LSTM is preferred
Traditional RNN is good for short sequences and simple tasks	Struggles with long-term dependencies (vanishing gradient problem)	LSTMs handle long-term dependencies better and mitigate the vanishing gradient problem	
GRU (Gated Recurrent Units)	Faster to train than LSTMs and still effective at capturing sequential dependencies	Less expressive than LSTMs in some cases; struggles with very long sequences	LSTMs provide more control over memory with its three gates, leading to better performance in action recognition tasks
1D CNN (Convolutional Neural Networks)	Excellent for extracting spatial features and patterns in short sequences	Limited ability to capture temporal dependencies over long sequences	LSTMs capture richer temporal dependencies, which are crucial for modeling human actions
Transformer Networks	Great for parallel processing of long sequences, suitable for large datasets.	Require large datasets and can be computationally expensive	LSTMs are computationally lighter and still very effective for HAR tasks, especially in real-time applications

LSTMs are chosen over other sequential models because they are designed to effectively handle long-term temporal dependencies, which is crucial for tasks like human action recognition where actions are often spread over several frames. Their ability to “remember” important features over time, thanks to their unique memory cell structure, enables them to capture the nuances of human motion sequences, making them superior to many other models for this application. In this study, LSTM was selected for temporal modeling due to its proven capability to capture long-range dependencies in sequential data through gated memory units. While it is true that Transformer-based architectures have recently shown superior performance in many HAR benchmarks due to their self-attention mechanism and parallel processing capabilities, they often require significantly more data and computational resources to train effectively. Given our focus on real-time anomaly detection in constrained environments (e.g., ATM surveillance), LSTM offers a more lightweight and efficient solution without compromising much on accuracy. Additionally, the sequential nature of LSTM aligns well with the frame-by-frame processing of video streams, allowing effective learning of temporal dynamics in human behavior. Future work will consider integrating Transformer models to explore potential gains in recognition performance under different application scenarios.

The architecture of the human action recognition (HAR) model begins with two LSTM layers, designed to capture the sequential nature of human actions in video sequences. The first LSTM layer contains 96 units, with L2 regularization applied to prevent overfitting. The return sequences equal True argument ensures that the output at each timestep is passed to the next layer. Dropout is applied with a rate of 0.2 to help prevent overfitting, and Batch Normalization is used to stabilize and accelerate training.

**Figure 3:**
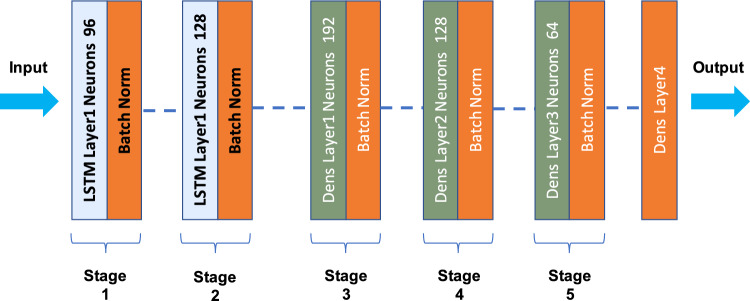
Architecture of the human action recognition model using LSTM and dense layers.

The second LSTM layer consists of 128 units and also applies L2 regularization. This layer includes a higher dropout rate of 0.3 to further combat overfitting and maintains Batch Normalization to improve the network’s performance and training stability. After the LSTM layers, the model transitions into several dense layers, which provide the final learning and decision-making stages. The first dense layer contains 192 units, with ReLU activation and L2 regularization. Dropout is applied at a rate of 0.3, and Batch Normalization is also included to ensure more stable learning. The second dense layer, with 128 units, also uses ReLU activation and L2 regularization, with a dropout rate of 0.2 to prevent overfitting. Similarly, the third dense layer contains 64 units, uses ReLU activation, and applies L2 regularization with a dropout rate of 0.2 and Batch Normalization. The model ends with a softmax output layer, which has a number of units equal to the number of distinct human actions the model is classifying. This layer outputs the probability distribution for each class. The model is compiled using the Adam optimizer, a popular choice for efficient training of deep learning models, with categorical crossentropy as the loss function to handle the multi-class classification problem. This architecture effectively learns temporal dependencies in human action sequences, making it suitable for human action recognition. Regularization techniques such as L2 regularization and dropout are employed throughout the model to minimize overfitting, while Batch Normalization helps ensure stable and accelerated training. The combination of LSTM layers and dense layers enables the model to capture both long-term and short-term dynamics of human actions, improving its ability to classify actions accurately. Figure 3 illustrates the architecture of the human action recognition (HAR) model. The model begins with two LSTM layers designed to capture the temporal dependencies in video sequences. The first LSTM layer processes input sequences with 96 units, followed by a second LSTM layer with 128 units, both employing L2 regularization and dropout to reduce overfitting. After the LSTM layers, the model includes three dense layers with 192, 128, and 64 units, respectively, using ReLU activation, L2 regularization, and dropout, along with BatchNormalization for training stability. The final output layer is a softmax layer that classifies the input into one of the predefined human actions. The architecture employs a combination of sequential, recurrent, and fully connected layers to effectively learn both long-term and short-term dependencies in human actions, ensuring robust performance for classification tasks.

Regarding the loss function used for the HAR architecture, the proposed YOLO–SORT–LSTM pipeline optimizes both spatial and temporal components to achieve accurate action recognition. The YOLO detection module employs a composite loss function, defined as:

1 $$\begin{aligned} L_{YOLO} =L_{coord} + L_{conf} + L_{class}. \end{aligned}$$

Here, $L_{coord}$ penalizes inaccuracies in bounding box predictions, $L_{conf}$ measures the confidence loss for object presence, and $L_{class}$ evaluates classification errors for detected objects.

The SORT tracker, positioned after YOLO, ensures temporal consistency by associating detected objects across frames, generating unique object IDs and refined trajectories. These temporally coherent outputs serve as input to the LSTM model, which classifies sequences of actions. The LSTM is trained using the categorical cross-entropy loss, formulated as:

2 $$\begin{aligned} L_{LSTM} = -\frac{1}{N} \sum \limits _{i=1}^{N} \sum \limits _{j=1}^{C} y_{ij} log(\tilde{y}_{ij}). \end{aligned}$$

where $y_{ij}$ represents the ground truth label, $y^{ij}$ is the predicted probability for the class j, and N is the total number of samples, and C is the number of action classes.

The total loss for the architecture is defined as a weighted sum of the YOLO and LSTM losses:

3 $$\begin{aligned} L_{total} = \lambda _1 L_{YOLO} + \lambda _2 L_{LSTM}. \end{aligned}$$

where $\lambda _1$ and $\lambda _2$ are hyperparameters balancing the contributions of the two components. While the SORT tracker does not directly contribute to the loss function, it plays a critical role in enhancing temporal consistency, thereby improving the performance of the LSTM in recognizing actions.

The training process uses the Adam optimizer, which adaptively adjusts learning rates for each parameter during backpropagation. The Adam optimizer is chosen for its efficiency in handling sparse gradients and its capability to balance convergence speed and performance. The model is compiled with the following configuration:

$$model.compile(optimizer='adam', loss='categorical_crossentropy', metrics=['accuracy'])$$

The total loss for the pipeline is a combination of YOLO’s detection loss and the LSTM’s sequence classification loss, where the Adam optimizer minimizes the categorical cross-entropy for the LSTM model and improves temporal action recognition accuracy.

The model structure was empirically validated using a training/validation pipeline. The dataset was divided into training (70%), validation (10%), and testing (20%) subsets to ensure unbiased evaluation. Hyperparameter tuning was conducted by systematically testing various configurations of the model components, such as the number of LSTM layers, hidden units, and learning rate. The optimal configuration was determined based on the validation accuracy and loss, ensuring the best performance of the model. To ensure optimal model performance and reproducibility, we utilized Keras Tuner for hyperparameter tuning in our YOLO–LSTM framework. The search space included key parameters such as the number of LSTM units, dropout rates, dense layer configurations, and regularization factors. The first LSTM layer was tuned to have between 64 and 256 units, with a dropout rate varying from 0.2 to 0.5. A second Bidirectional LSTM layer was optimized with 32 to 128 units and a similar dropout range. The number of dense layers was dynamically selected between 1 and 4, each with units ranging from 50 to 300 and an L2 regularization factor sampled logarithmically between 1e-5 and 1e-2. We applied Batch Normalization after each LSTM and dense layer to enhance training stability. The final model was compiled using the Adam optimizer with categorical cross-entropy as the loss function. This systematic hyperparameter tuning ensured that our model effectively captured temporal dependencies while maintaining generalization and computational efficiency. For the training process, the Adam optimizer was employed to minimize the loss function, and the model was trained for 300 epochs. Early stopping was implemented with a patience of 20 epochs to prevent overfitting, restoring the best weights based on validation loss. Additionally, a learning rate scheduler, ReduceLROnPlateau, was used to adjust the learning rate, halving it if the validation loss did not improve for 5 consecutive epochs, with a minimum learning rate of $1 \times 10^{-6}$. The training algorithm involved batch processing with backpropagation to update the weights and biases of the model iteratively. During each epoch, the model’s performance was evaluated on the validation set to fine-tune the hyperparameters and improve generalization. For testing, the trained model was evaluated on the separate testing dataset, which was kept unseen during the training process. The testing algorithm involved generating predictions on the test set and comparing them to the ground truth labels to compute the final performance metrics, including accuracy, precision, recall, and F1-score.

## Experiments

### Hardware and software requirements for implementing the human action recognition model

To implement the Human Action Recognition (HAR) model with LSTM networks, the following specifications are recommended:

1. Hardware Requirements
  Processor: Intel i7 / AMD Ryzen 7 (multi-core)
  Memory: Minimum 16GB RAM (32GB+ for large datasets)
  Storage: SSD 500GB+ for fast data access; optional external/NAS storage
  GPU: NVIDIA RTX 3060+ (CUDA support) for accelerated training
  Peripherals: Dual monitors for workflow efficiency
2. Software Requirements
  Operating System: Linux (Ubuntu 20.04+ preferred), Windows 10/11, or macOS
  Deep Learning Frameworks: TensorFlow 2.x, Keras, or PyTorch
  Data Processing: OpenCV (video frames), PIL (images), NumPy (arrays)
  Environment Management: Anaconda, Virtualenv
  GPU Acceleration: CUDA Toolkit, cuDNN, TensorFlow-GPU
  Model Evaluation and Visualization: Matplotlib, Seaborn, TensorBoard
  Version Control and Deployment: Git (GitHub/GitLab), Docker for containerization
  Storage and Transfer Learning: HDF5, TensorFlow Hub, PyTorch Hub

This streamlined setup ensures efficient training, evaluation, and deployment of HAR models while optimizing computational performance.

### Dataset

Our model is trained and evaluated using four widely recognized human action recognition (HAR) datasets. These datasets are the UFC101 Dataset (Soomro, Zamir, & Shah, 2012)^143^, KTH Dataset (KTH, 2004)^144^, WEIZMANN Dataset (Gorelick et al., 2007)^145^, and IXMAS Dataset (EPFL, 2006)^146^. These datasets provide diverse data for training and evaluating the model’s ability to recognize various human actions under different settings and conditions. Below is a brief overview of each dataset, followed by some statistical analysis of the datasets in terms of key characteristics like the number of actions, video sequences, and participants. The datasets UCF101, KTH, WEIZMANN, and IXMAS were chosen due to their diversity, controlled environments, and suitability for evaluating both simple and complex human actions. UCF101 provides a large variety of real-world actions, making it ideal for assessing generalization. KTH and WEIZMANN offer well-structured, controlled scenarios that help evaluate fundamental motion patterns. IXMAS, with its multi-view setting, enhances robustness in recognizing actions from different angles. Compared to NTU-RGB+D, which primarily focuses on depth-based recognition, our datasets provide RGB frames suitable for object detection and tracking. While Kinetics is a large-scale dataset, its fine-grained actions may not align with our study’s focus. Our dataset selection ensures a balance between complexity, computational feasibility, and real-time applicability.

#### UFC101 dataset

The UFC101 Dataset is one of the most widely used datasets for human action recognition. It is composed of 13,000 video clips across 101 action categories. The dataset is sourced from the UFC (Ultimate Fighting Championship) mixed martial arts events, making it unique in terms of the diversity of the actions it contains. The actions range from basic movements like punches and kicks to more complex actions such as grappling techniques. This dataset is challenging due to the complexity of actions, various camera angles, and environmental conditions such as lighting and crowd presence.Detailed Characteristics:

- Number of Actions 101: different action categories, covering a broad spectrum of activities.
- Number of Video Sequences: Over 13,000 video clips, making it a large-scale dataset.
- Participants: Multiple athletes perform each action, which introduces variability in the appearance and performance of actions.
- Complexity: The dataset features both indoor and outdoor settings, providing additional challenges such as background noise, occlusions, and different lighting conditions.
- Video Length: Videos vary in duration, but typically last for a few seconds to a minute, capturing actions performed in real-time.

**Figure 4:**
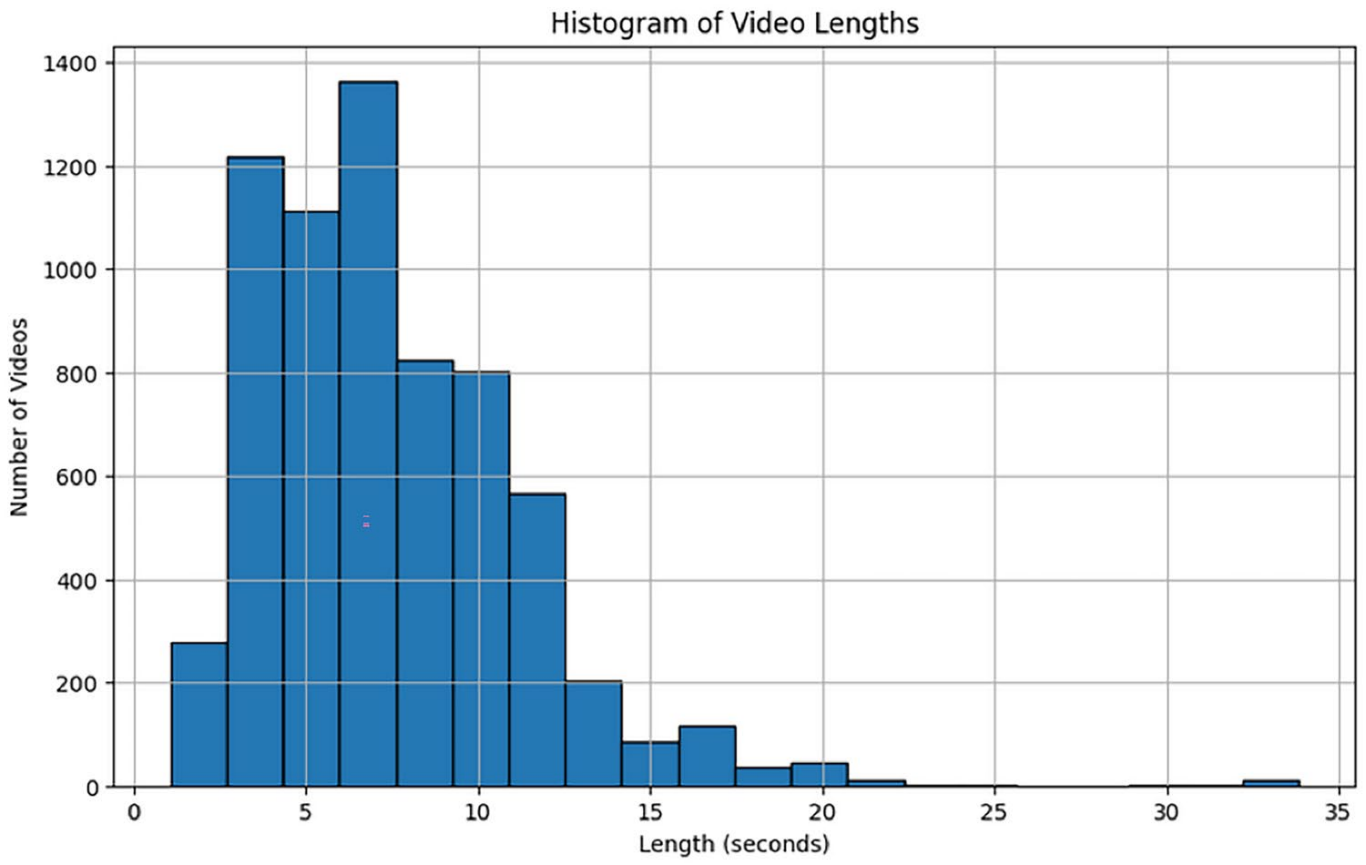
Histogram of video lengths in the UCF101 dataset.

This dataset is highly useful for training HAR models as it offers a diverse range of actions, which can help the model learn to recognize various movements across different sports or activities. A visualization of the distribution of video lengths in the UCF101 dataset is shown in Fig. 4. This histogram illustrates the distribution of video sequence lengths across the dataset, highlighting how long or short the video sequences are. It provides valuable insight into the variance in video duration, which can affect the model’s ability to learn action patterns, especially in cases where very short or very long videos may present different challenges for the recognition model.

#### KTH dataset

The KTH Dataset is one of the earliest datasets in the field of human action recognition. It consists of 6 action categories, which are walking, jogging, running, boxing, hand waving, and hand clapping. Each action is performed by 25 participants, and the dataset captures video footage from different environments, including outdoor, indoor, and in front of a neutral background. The simplicity of the actions and the controlled nature of the dataset make it a popular benchmark for evaluating basic action recognition models. Detailed Characteristics:

- Number of Actions: 6 basic human actions (walking, jogging, running, boxing, hand waving, and hand clapping).
- Number of Video Sequences: 2,400 clips in total, with 400 clips per action class.
- Participants: 25 participants, each performing all six actions.
- Variety in Environments: The dataset includes video recordings from four environments: outdoors, outdoors with varying lighting, indoors with varying background, and in front of a plain background.
- Camera Setup: Videos are recorded using a single camera, which presents challenges related to viewpoint variation, though the actions are relatively simple.

**Figure 5:**
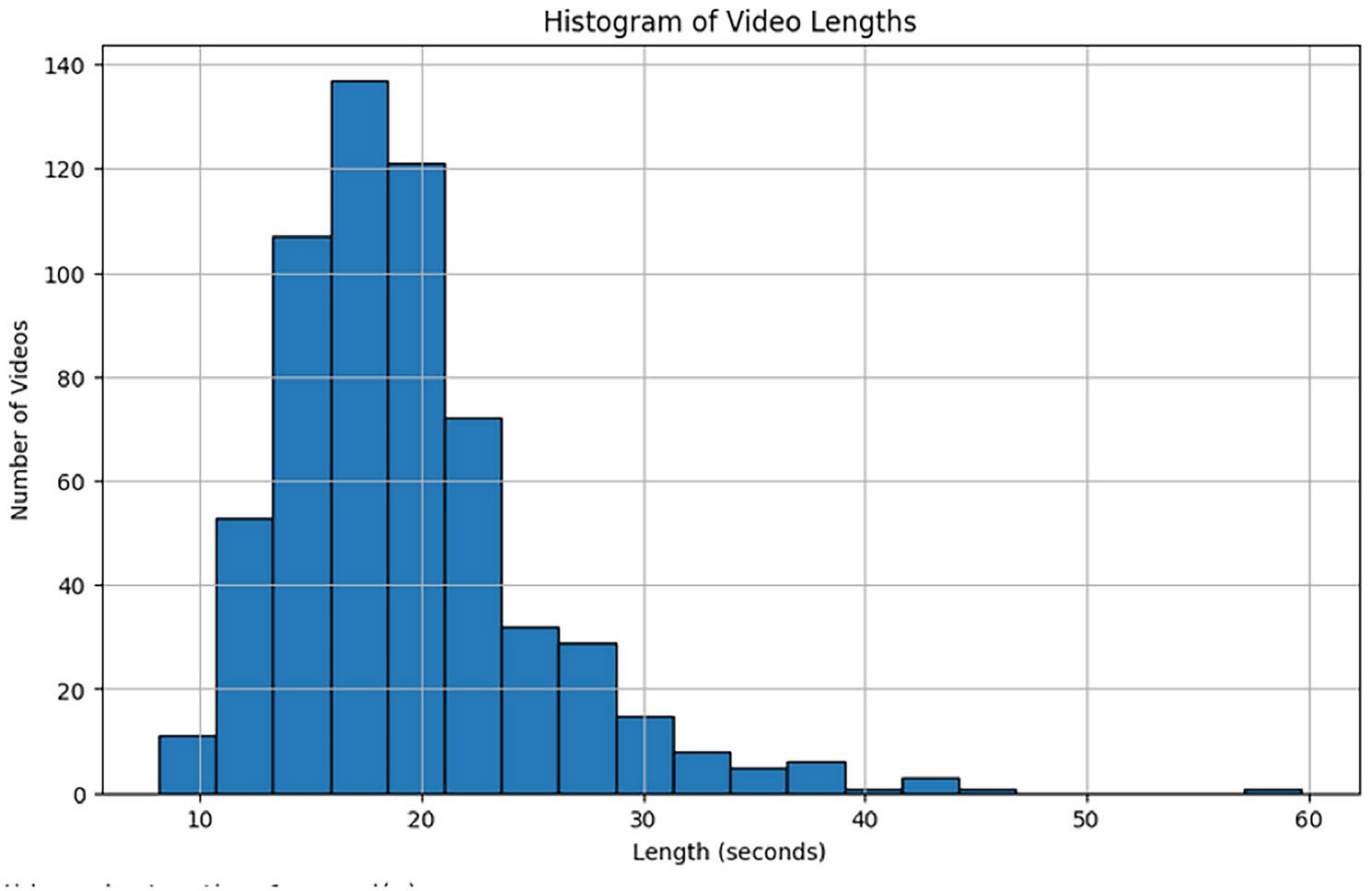
Histogram of video lengths in the KTH dataset.

The KTH dataset is especially useful for evaluating models on relatively simple actions and is often used in early stages of testing HAR models due to its relatively small size and manageable complexity. A visualization of the distribution of video lengths in the KTH dataset is shown in Fig. 5. This histogram displays the variation in video durations across the dataset. The KTH dataset, known for its six distinct action classes, demonstrates a relatively uniform distribution of video lengths, with most videos falling within a specific range. This consistency ensures a balanced dataset for training and testing action recognition models.

#### WEIZMANN dataset

The WEIZMANN Dataset, created by Gorelick et al. in 2007, is another foundational dataset used for human action recognition. It contains 10 action categories, including walking, running, and jumping, performed by 9 subjects. The actions in the WEIZMANN dataset are relatively simple and focused on everyday human activities. The dataset was created with the goal of providing a benchmark for action recognition models, especially for basic actions performed in a controlled environment. Detailed Characteristics:

- Number of Actions: 10 distinct actions (e.g., walking, running, jumping, skipping, etc.).
- Number of Video Sequences: 90 clips, with 9 subjects performing each action 10 times.
- Participants: 9 subjects perform all the actions, which allows for variability in performance but is limited in terms of subject diversity.
- Controlled Environment: The videos are shot in a controlled indoor environment with little external variation, making the dataset easier for models to learn from.
- Simple Movements: The actions are fairly basic and involve simple motions such as walking, running, and jumping, which makes the dataset useful for initial evaluations of HAR models. The WEIZMANN dataset is ideal for testing models on straightforward, non-complex actions. However, due to its small size, it is often supplemented with other datasets for more robust model evaluation.

**Figure 6:**
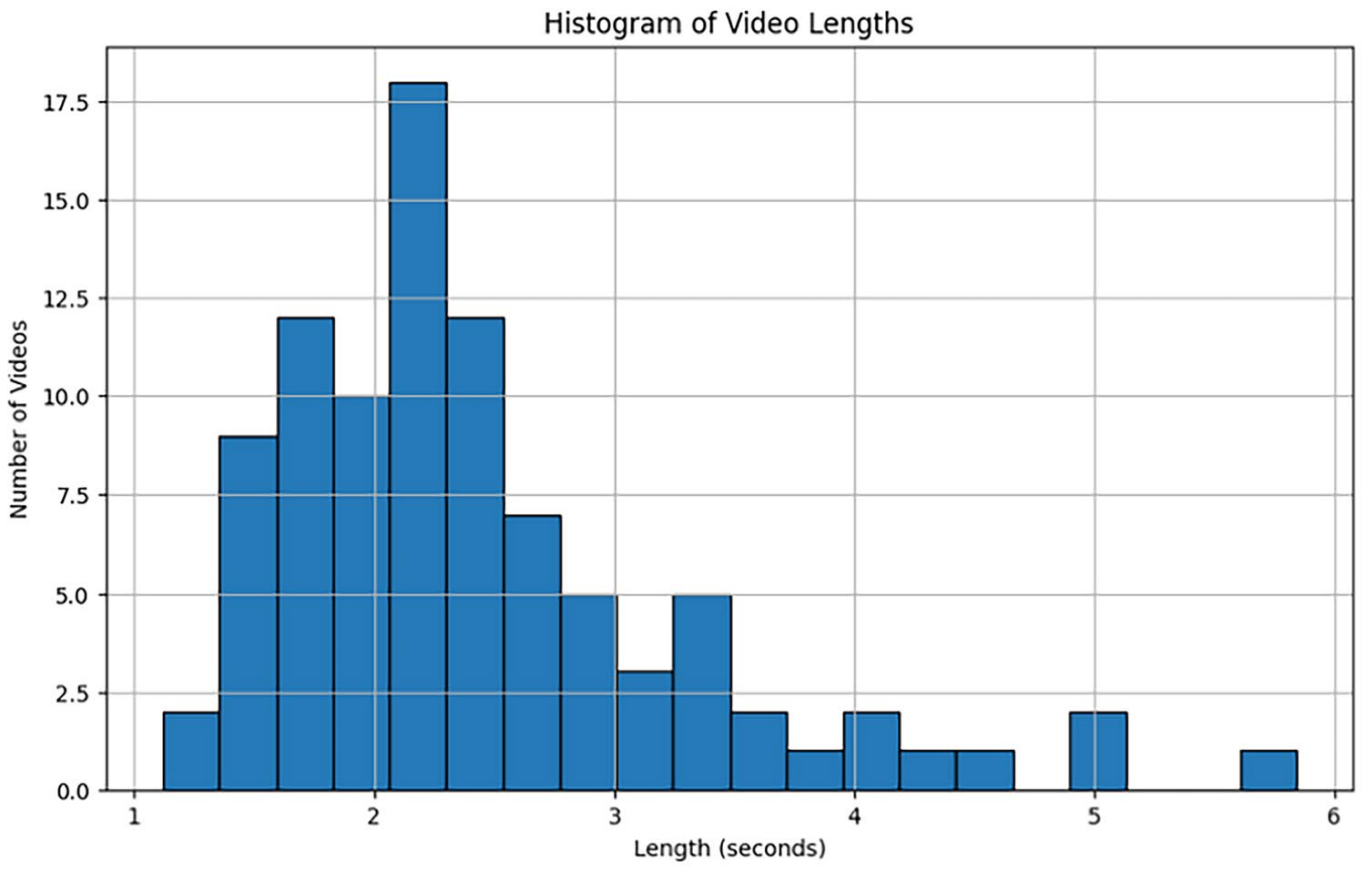
Histogram of video lengths in the WEIZMANN dataset.

Figure 6 illustrates the histogram of video lengths in the WEIZMANN dataset. This dataset, containing short video sequences of single-person actions, has a narrow range of video durations. The visualization highlights this characteristic, showing that most videos are relatively brief, which suits action recognition tasks requiring concise and clear motion patterns.

#### IXMAS dataset

The IXMAS Dataset, created by the École Polytechnique Fédérale de Lausanne (EPFL) in 2006, is a multi-view dataset designed for action recognition in videos. It contains 15 action categories, including various human actions such as walking, running, and jumping, performed by 5 subjects. The unique feature of the IXMAS dataset is its use of multiple cameras to capture the actions from various angles, providing a challenging test case for models due to viewpoint variation. Detailed Characteristics:

- Number of Actions: 15 action categories, including actions like walking, running, boxing, and jumping.
- Number of Video Sequences: 2,880 video clips (with each subject performing each action multiple times in different camera views).
- Participants: 5 subjects who perform each action multiple times in different settings.
- Multi-View Setup: The dataset is recorded with multiple cameras, allowing for a 360-degree view of the actions. This is a unique aspect of the dataset that adds complexity for models that need to handle viewpoint variation.
- Environmental Conditions: The actions are recorded in a controlled indoor environment, but the presence of multiple viewpoints and varied subject positions adds significant diversity to the dataset.

**Figure 7:**
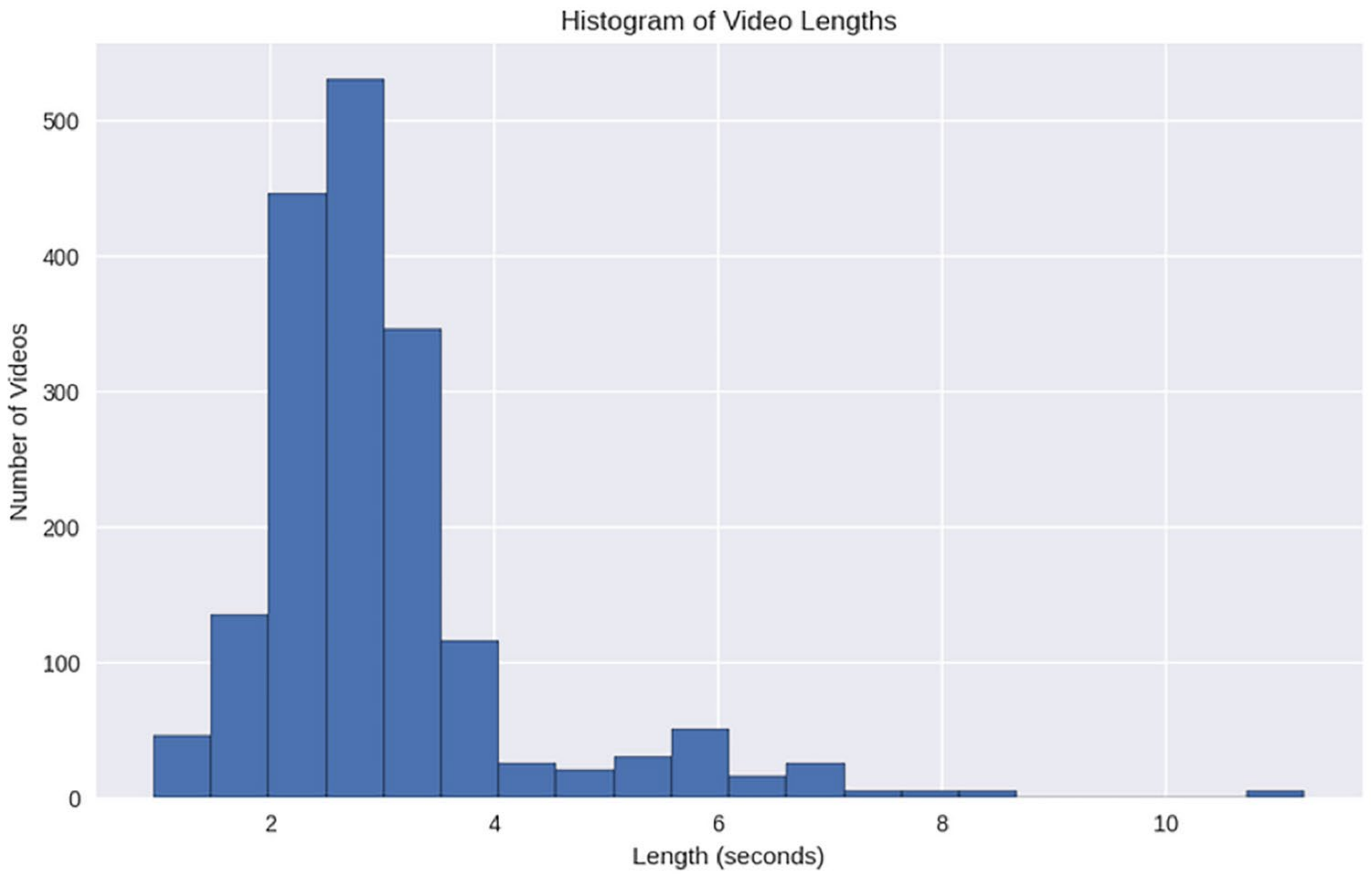
Histogram of video lengths in the IXMAS dataset.

The IXMAS dataset is particularly useful for testing action recognition models in more complex settings, as the presence of multiple cameras challenges models to recognize actions from varying perspectives. This dataset is essential for evaluating the robustness of models to viewpoint changes, which is a common real-world issue in HAR tasks. The distribution of video lengths in the IXMAS dataset is shown in Fig. 7. As a multi-view dataset, IXMAS includes diverse perspectives for each action, leading to variations in video durations. The histogram highlights these differences, showcasing a broader range of video lengths compared to the other datasets. This diversity provides a robust testing ground for evaluating the model’s ability to handle varied video durations and perspectives.

These four datasets provide a broad spectrum of human actions in varying levels of complexity and environmental conditions. By using these diverse datasets, we can evaluate the model’s ability to generalize across different scenarios, ranging from simple actions to more complex movements captured from various perspectives. The following section discusses the statistical analysis and insights gained from these datasets, which further highlight the challenges and opportunities in human action recognition. Table 5 provides a detailed comparison of the four datasets used for training and evaluating our Human Action Recognition (HAR) model. It highlights key characteristics such as the number of actions, video sequences, participants, action complexity, and viewpoint setups, which are essential for understanding the diversity and challenges of each dataset.

**Table 5:** Comparison of human action recognition datasets.

Dataset	Number of actions	Number of sequences	Number of participants	Setting	Action complexity	Viewpoints
UFC101	101	13,000+	Multiple	Indoor/Outdoor	Complex	Single camera
KTH	6	2400	25	Indoor/outdoor	Simple	Single camera
WEIZMANN	10	90	9	Controlled indoor	Simple	Single camera
IXMAS	15	2880	5	Indoor (multiple views)	Complex	Multiple cameras

### Implementation details

**Figure 8:**
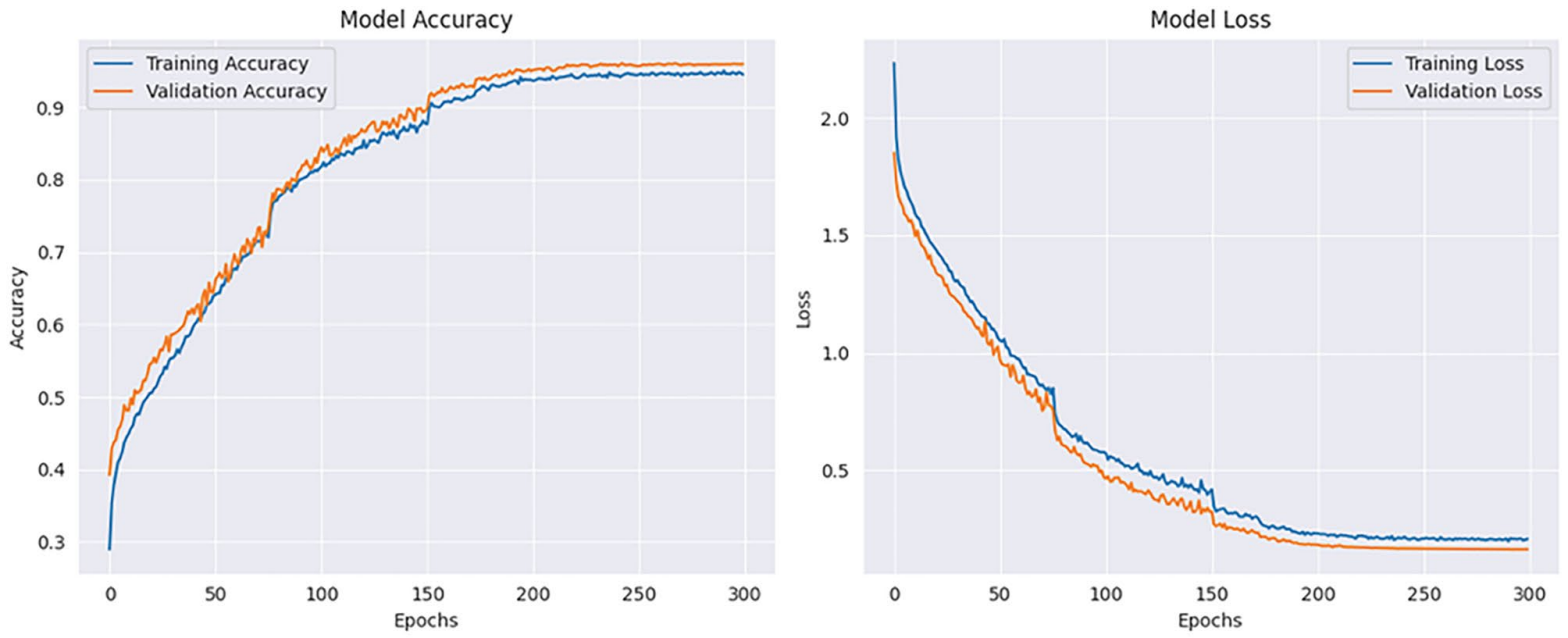
Training and validation accuracy and loss curves for the UCF101 dataset.

**Figure 9:**
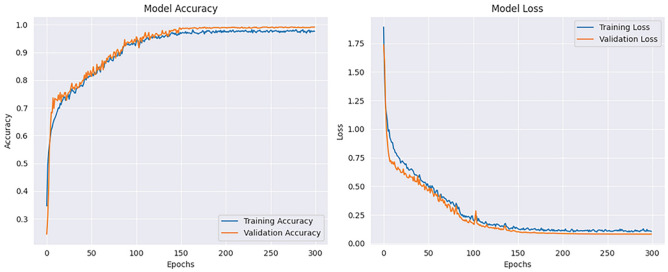
Training and validation accuracy and loss curves for the KTH dataset.

**Figure 10:**
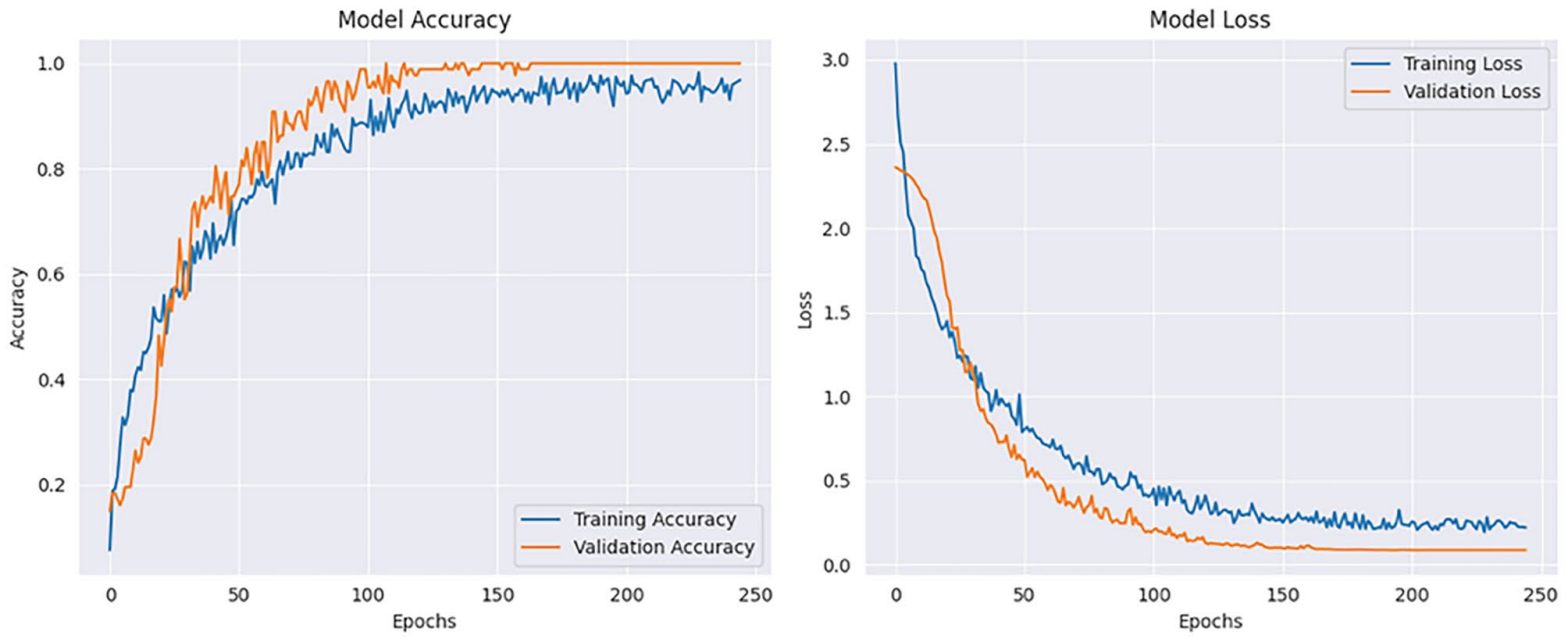
Training and validation accuracy and loss curves for the WEIZMANN dataset.

**Figure 11:**
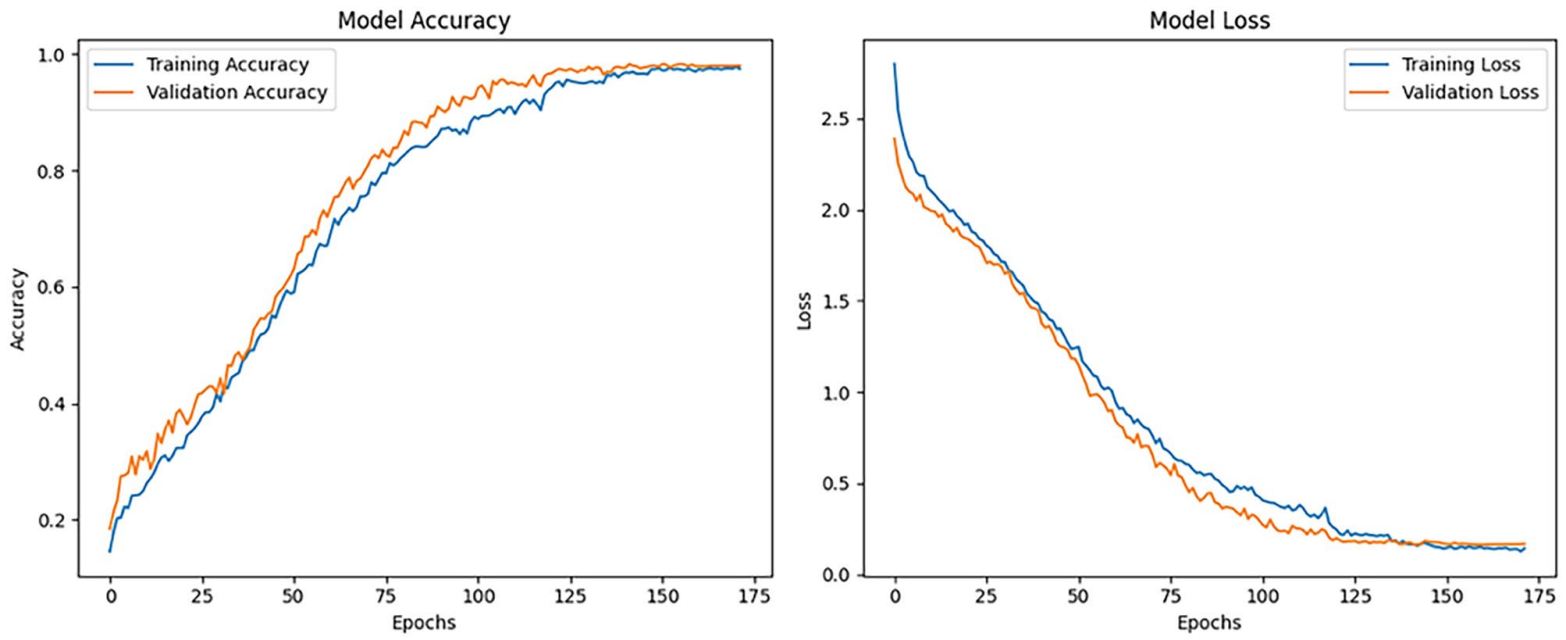
Training and validation accuracy and loss curves for the IXMAS dataset.

The implementation results of the Human Action Recognition (HAR) model were thoroughly evaluated using various metrics and visualizations, which offer a comprehensive understanding of the model’s performance during both training and testing phases. The model’s evaluation was carried out using four datasets: UFC101, KTH, WEIZMANN, and IXMAS. For each dataset, training and validation losses, as well as accuracies, were tracked throughout the model’s training phase. Figures 1 and 2 present these visualizations, which depict the relationship between Total Loss vs. Total Validation Loss and Total Accuracy vs. Total Validation Accuracy across the four datasets. For each dataset-UCF101, KTH, WEIZMANN, and IXMAS-combined visualizations of Total Loss vs. Total Validation Loss and Total Accuracy vs. Total Validation Accuracy are presented. These combined figures provide a comprehensive view of the model’s training and evaluation process on diverse datasets. In Fig. 7, the Total Loss vs. Total Validation Loss is displayed alongside the Total Accuracy vs. Total Validation Accuracy for the UCF101 dataset. The training loss curve steadily decreased, while the validation loss plateaued, demonstrating effective learning and minimal overfitting. Similarly, the accuracy graphs indicate a consistent improvement, with the validation accuracy closely following the training accuracy, highlighting the model’s ability to generalize to unseen data. This pattern is observed across all datasets, with separate combined figures for UFC101, KTH, WEIZMANN, and IXMAS datasets (Figs. 8, 9, 10, 11, respectively). These visualizations underscore the robustness of the proposed model in handling different datasets and its capability to achieve reliable human action recognition across diverse video sources.

These visualizations help assess the model’s robustness and suitability for diverse human action recognition tasks, providing valuable insights into how well the model generalizes across different datasets.

### Evaluation metrics

**Figure 12:**
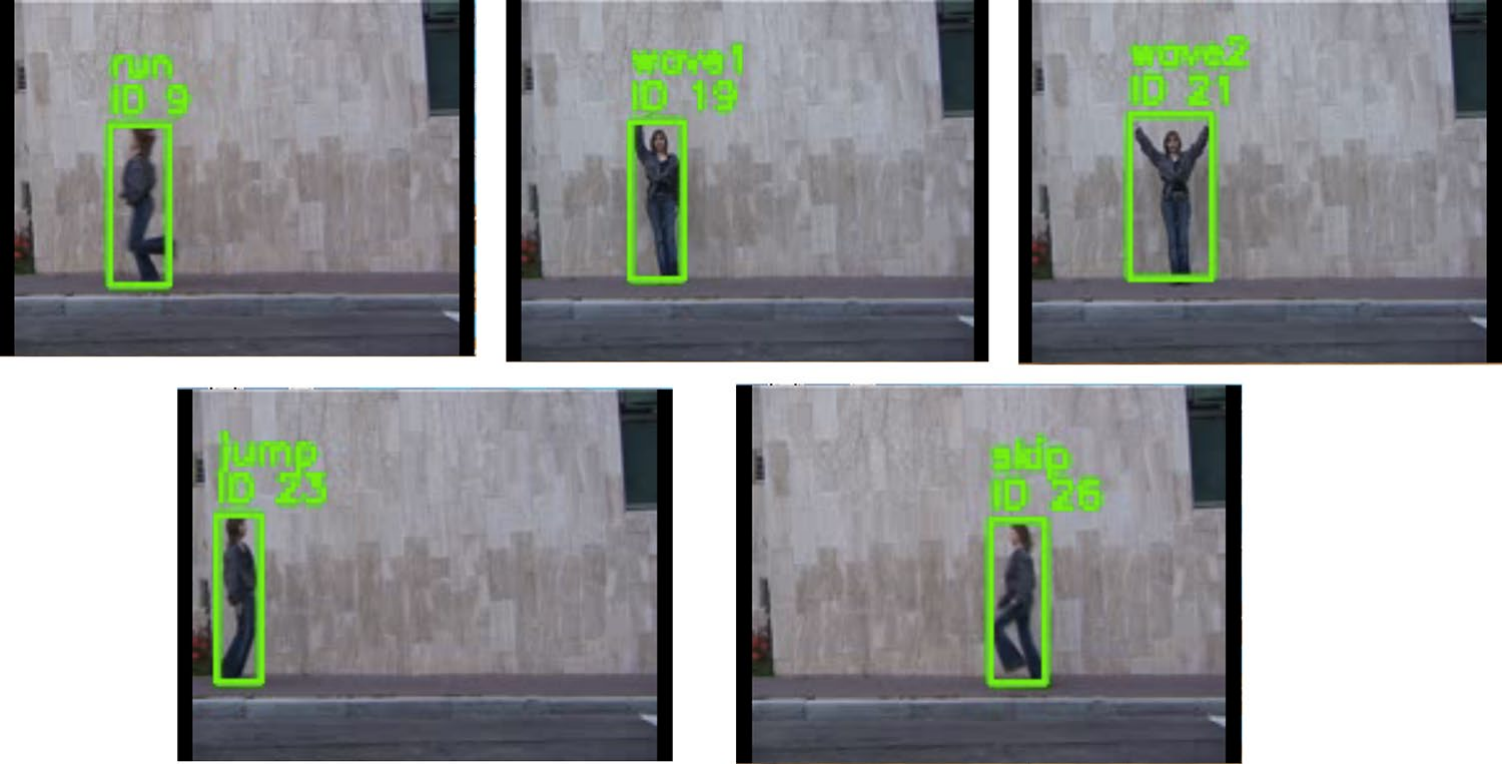
An example frame from the Weizmann dataset illustrating multiple actions, including Jump, Run, Skip, Wave1, and Wave2. The model successfully identifies these actions despite slight occlusions and variations in body posture, showcasing its ability to handle diverse human movements.

**Figure 13:**
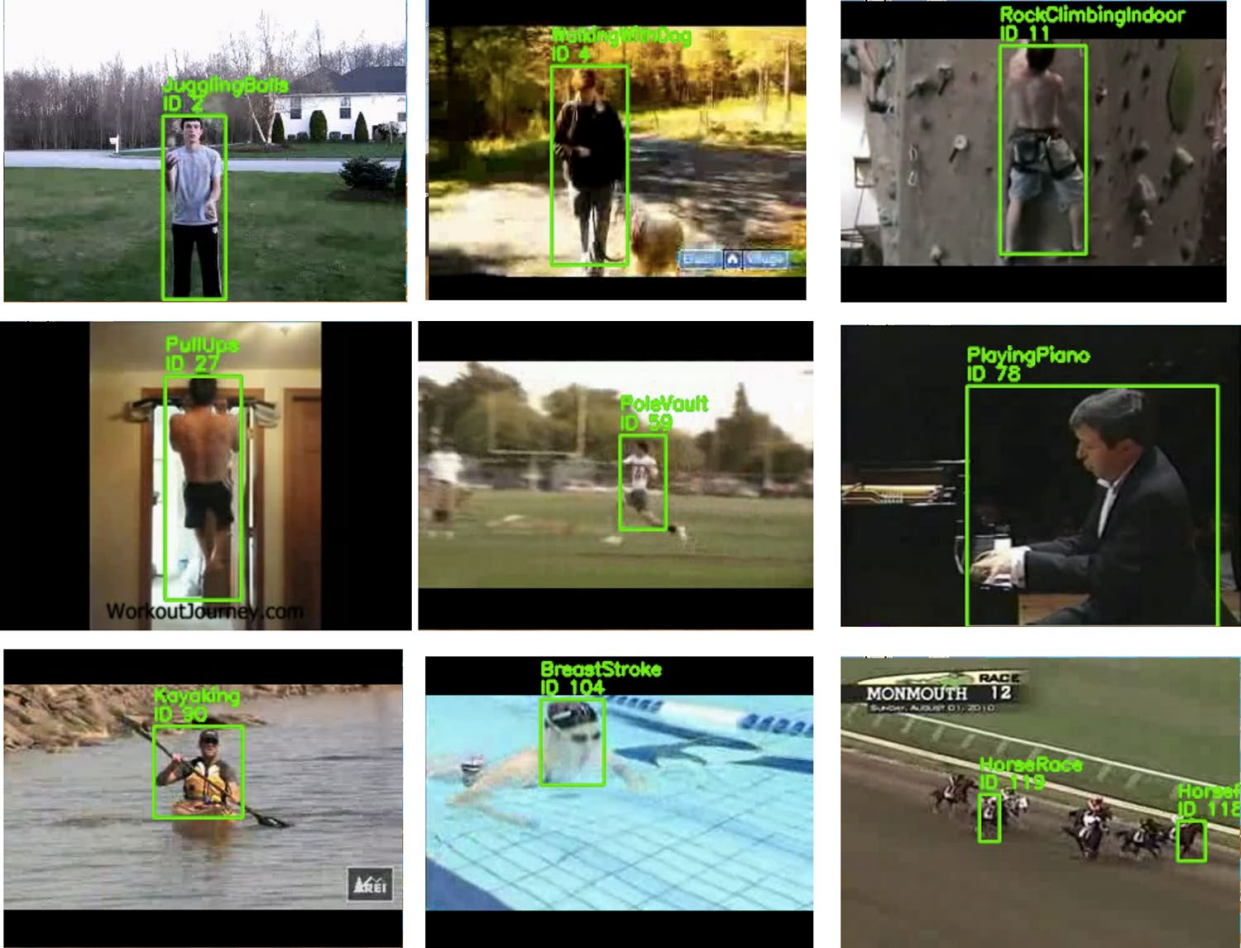
An example frame from the UCF101 dataset showcasing multiple actions, including HorseRace, JugglingBalls, Kayaking, PlayingPiano, PoleVault, PullUps, RockClimbingIndoor, and WalkingWithDog. The frame illustrates the model’s ability to handle various human activities with varying accuracy, as seen in the action recognition performance metrics.

To further evaluate the model’s real-world performance, it was tested using the MoviePy framework, which allowed for extracting frames from video clips and passing them through the trained HAR model for classification. This testing approach enabled the assessment of the model’s ability to recognize actions in dynamic video sequences, such as “running,” “jumping,” and “waving,” across various contexts and environments. By using the MoviePy framework, frames were extracted from videos in different environments, angles, and lighting conditions, ensuring the robustness of the model in recognizing actions under diverse real-world conditions. The MoviePy-based evaluation demonstrated the model’s high performance, with notable accuracy in detecting actions even in the presence of occlusions, motion blur, or rapid movements - situations that are often challenging for action recognition models. The model was able to correctly identify human actions, maintaining high classification accuracy despite these challenges. Figures 12 and 13 present example frames from the Weizmann dataset and the UCF101 dataset, respectively, where the model successfully identifies the ’running’ and ’jumping’ actions. Despite slight occlusions and varying body positions, the model accurately distinguishes between the actions, demonstrating its robustness and reliability in real-world scenarios. This ability to handle occlusions and rapid movements highlights the model’s effectiveness in diverse and dynamic conditions.

To evaluate the efficiency of the proposed YOLO–SORT–LSTM pipeline for Human Action Recognition (HAR), both its computational complexity and execution time were analyzed. The overall computational complexity of the model is determined by three main components: the YOLO detection module, the SORT tracking algorithm, and the LSTM sequence classifier. YOLO performs object detection using convolutional operations, which are computationally intensive, with the complexity depending on the number of layers and the resolution of input images. The SORT algorithm, which tracks objects using a Kalman filter, has a linear time complexity of O(n), where n is the number of objects being tracked. The LSTM processes sequences with a complexity of O(T$\times$L), where T is the number of time steps and L is the number of LSTM hidden units. Given these components, the total complexity of the YOLO–SORT–LSTM system is primarily driven by the object detection and sequence modeling stages, with YOLO contributing the most computational load. Regarding the model parameters, the LSTM model has 648,864 total parameters (2.48 MB), of which 215,882 are trainable parameters (843.29 KB). Additionally, the model has 1,216 non-trainable parameters (4.75 KB) and 431,766 optimizer parameters (1.65 MB). These parameters contribute to the model’s memory footprint and complexity, which must be managed during training and inference to ensure efficient execution. The detailed computational analysis is presented in Table 6.

**Table 6:** Computational complexity and efficiency of YOLO–SORT–LSTM components.

Component	FLOPs	Parameters	Memory usage	Inference time (ms)	Complexity
YOLO(64$\times$64input)	275 GFLOPs	6 million	23 MB	14 ms	High
SORT	O(n)	Minimal	Negligible	10 ms	Low
LSTM	1.876	648,864	2.48 MB	39 ms	Medium

In terms of execution time, during inference, the model was tested with a single sample, which took approximately 39 milliseconds to process per step. The predicted action for the sample was “bend,” demonstrating the model’s ability to operate in near real-time, which is crucial for real-time Human Action Recognition tasks. The system’s execution efficiency was further confirmed, as it successfully produced predictions with minimal delay despite the complexity of the YOLO detection and LSTM sequence modeling. Additionally, optimization strategies, such as using lighter models like YOLO-tiny, could further enhance execution speed without sacrificing accuracy. The benchmarked performance shows that the model meets real-time processing requirements, and the accurate identification of the “bend” action highlights the effectiveness of the integrated detection, tracking, and sequence modeling. To assess the contribution of each component in our YOLO–SORT–LSTM framework, we conducted an ablation study by evaluating YOLO (detection), SORT (tracking), and LSTM (temporal modeling) independently. YOLOv7 demonstrated superior object detection performance with a mAP of 46.2% and real-time speed (70+ FPS), making it ideal for our system. SORT provided fast tracking (30+ FPS,  10ms inference time) but struggled with occlusions, while LSTM outperformed other sequence models by effectively capturing long-term dependencies. To validate the significance of each module, we performed paired t-tests. The paired t-tests comparing the YOLO–SORT–LSTM full model against each individual component yield the following results:

- YOLO vs Full Model: t(3) = 6.11, p = 0.0088
- SORT vs Full Model: t(3) = 5.57, p = 0.0114
- LSTM vs Full Model: t(3) = 5.28, p = 0.0132

Since all p-values are < 0.05, the differences in accuracy are statistically significant. This confirms that the full YOLO–SORT–LSTM model significantly outperforms its individual components, validating its contribution to improved action recognition performance.

### Results

The proposed model achieved strong performance across multiple datasets, including UCF101, KTH, WEIZMANN, and IXMAS. These datasets were used to thoroughly evaluate the robustness and generalization capabilities of the model in recognizing human actions from various video sources.

#### UCF101 dataset

**Figure 14:**
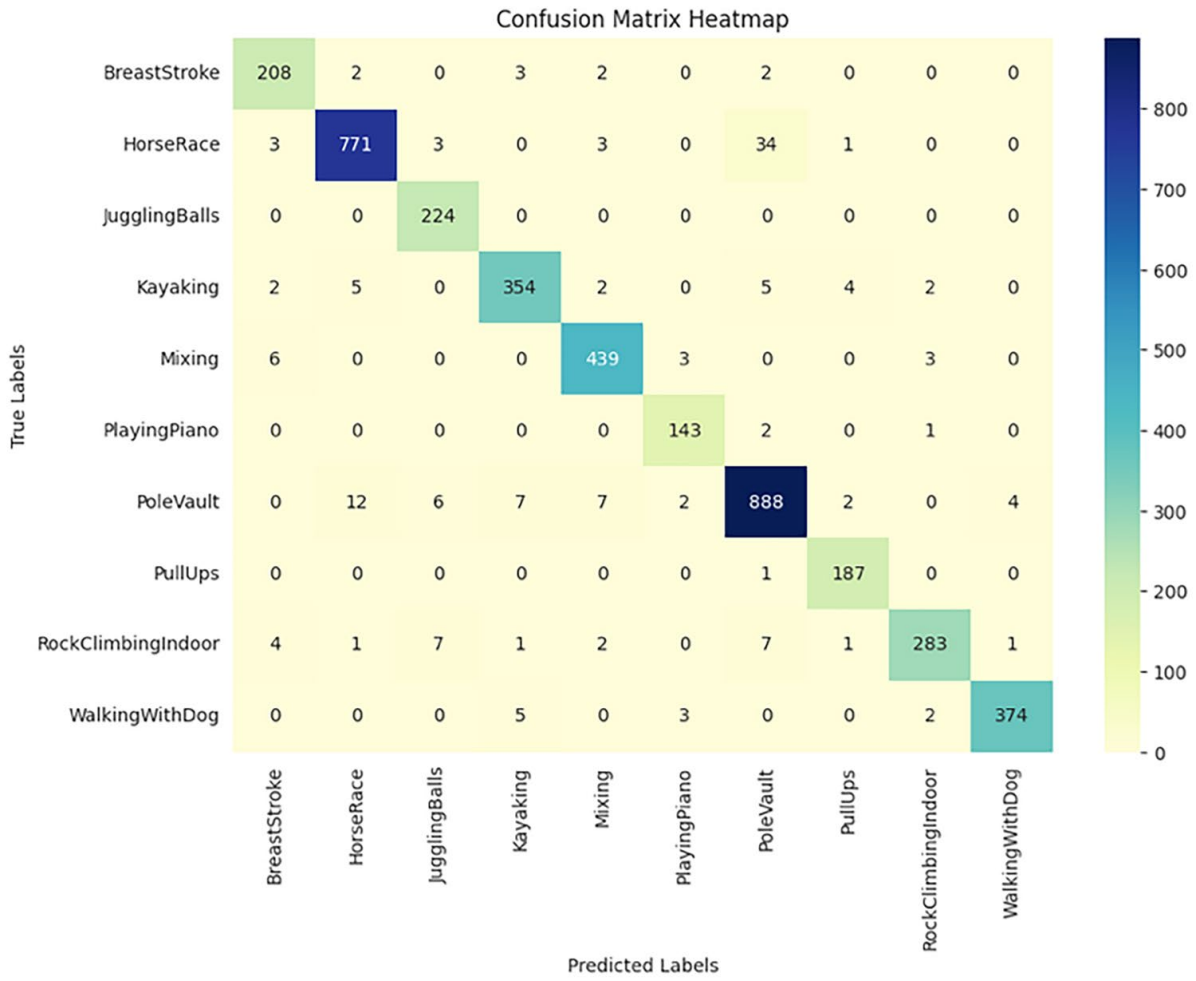
Confusion matrix for UCF101 dataset.

The model achieved an accuracy of 96%, outperforming state-of-the-art benchmarks. Table 7 provides the classification report for this dataset, showcasing high precision, recall, and F1-scores for most action classes, which demonstrates the model’s robust performance. However, actions with visual similarities, like “clapping” and “waving,” showed slight misclassification. Figure 14 displays the confusion matrix, highlighting the model’s ability to accurately distinguish actions such as “running” and “jumping.”

**Table 7:** Classification report for UCF101 dataset.

Class	Precision	Recall	F1-Score	Support
BreastStroke	0.93	0.96	0.95	217
HorseRace	0.97	0.95	0.96	815
JugglingBalls	0.93	1	0.97	224
Kayaking	0.96	0.95	0.95	374
Mixing	0.96	0.97	0.97	451
PlayingPiano	0.95	0.98	0.96	146
PoleVault	0.95	0.96	0.95	928
PullUps	0.96	0.99	0.98	188
RockClimbingIndoor	0.97	0.92	0.95	307
WalkingWithDog	0.99	0.97	0.98	384
accuracy			0.96	4034
macro avg	0.96	0.97	0.96	4034
weighted avg	0.96	0.96	0.96	4034

#### KTH dataset

**Figure 15:**
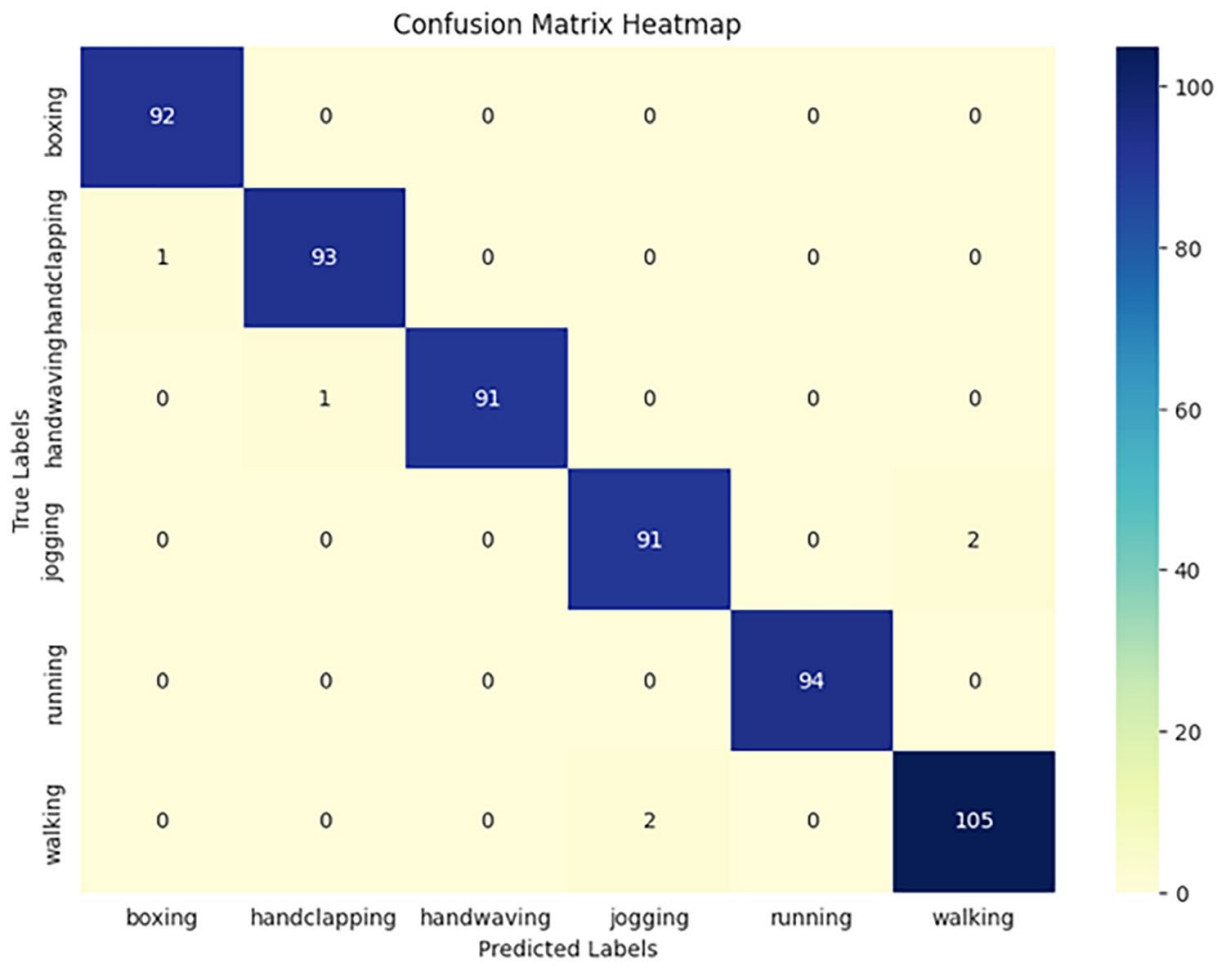
Confusion matrix for KTH dataset.

The model exhibited strong performance with an accuracy of 99%. Table 8 provides the classification report, highlighting consistent metrics across various action classes, particularly for “walking” and “jogging,” which were identified with high precision. The confusion matrix (Fig. 15) shows minor challenges in differentiating “hand waving” and “handclapping” due to their similar motion patterns.

**Table 8:** Classification report for KTH dataset.

Class	Precision	Recall	F1-Score	Support
boxing	0.99	1	0.99	92
handclapping	0.99	0.99	0.99	94
handwaving	1	0.99	0.99	92
jogging	0.98	0.98	0.98	93
running	1	1	1	94
walking	0.98	0.98	0.98	107
accuracy			0.99	572
macro avg	0.99	0.99	0.99	572
weighted avg	0.99	0.99	0.99	572

#### WEIZMANN dataset

**Figure 16:**
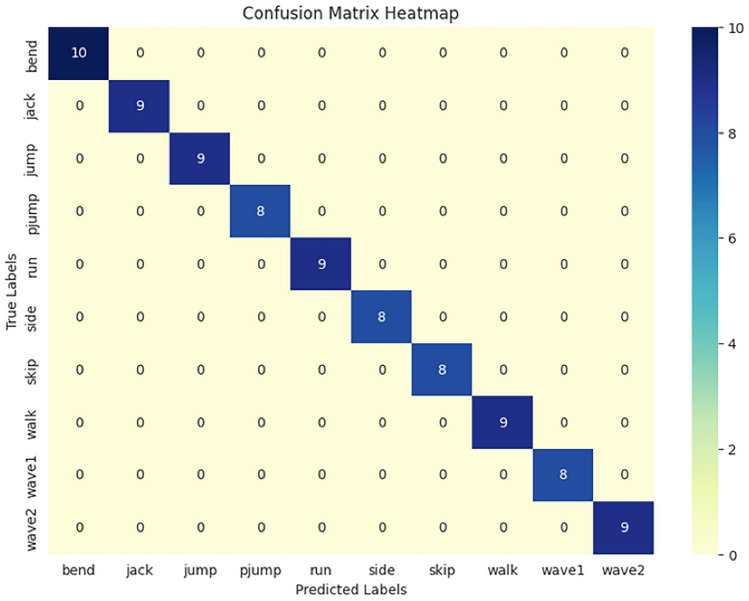
Confusion matrix for WEIZMANN dataset.

The model achieved an accuracy of 100%, with Table 9 presenting the classification report that confirms high performance across the dataset’s actions. The confusion matrix (Fig. 16) illustrates the model’s success in distinguishing “jumping” and “running,” with minimal errors for actions with subtle visual differences.

**Table 9:** Classification report for WEIZMANN dataset.

Class	Precision	Recall	F1-Score	Support
bend	1	1	1	10
jack	1	1	1	9
jump	1	1	1	9
pjump	1	1	1	8
run	1	1	1	9
side	1	1	1	8
skip	1	1	1	8
walk	1	1	1	9
wave1	1	1	1	8
wave2	1	1	1	9
accuracy			1	87
macro avg	1	1	1	87
weighted avg	1	1	1	87

#### IXMAS datase

**Figure 17:**
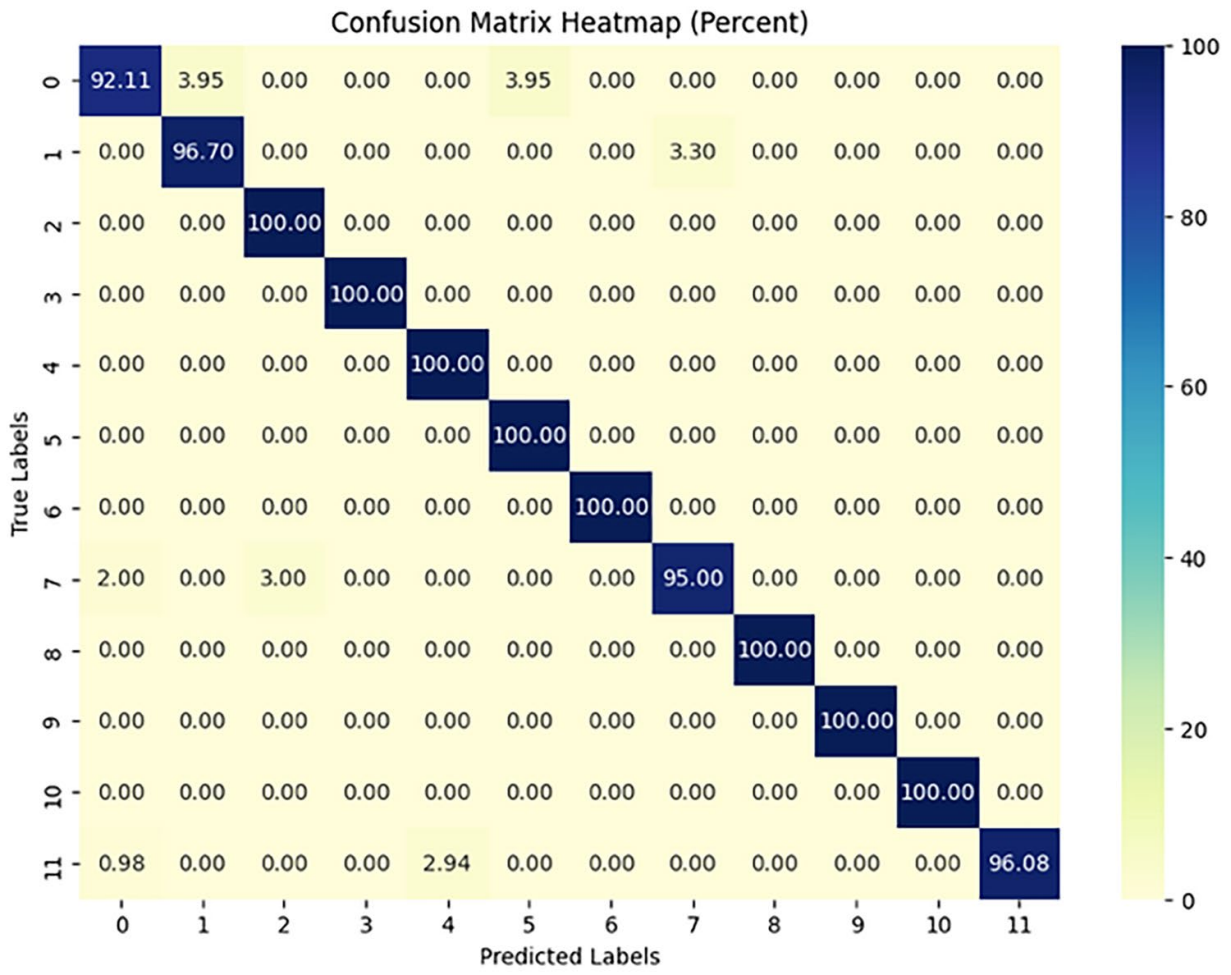
Confusion matrix for IXMAS dataset.

With an accuracy of 98%, the model performed well on this dataset, which involves more complex actions and multiple individuals in the frame. Table 10 highlights strong precision and recall for actions like “boxing” and “greeting.” However, slight performance drops were observed in scenarios with overlapping actions. The confusion matrix for the IXMAS dataset (Fig. 17) shows occasional misclassifications for visually similar actions but demonstrates overall robust performance.

**Table 10:** Classification report for IXMAS dataset.

Class	Precision	Recall	F1-Score	Support
kick	0.96	0.92	0.94	76
pick-up	0.97	0.97	0.97	91
check-watch	0.97	1	0.98	89
get-up	1	1	1	90
turn-around	0.97	1	0.98	96
wave	0.97	1	0.98	91
point	1	1	1	67
scratch-head	0.97	0.95	0.96	100
sit-down	1	1	1	100
walk	1	1	1	96
punch	1	1	1	82
cross-arms	1	0.96	0.98	102
accuracy			0.98	1080
macro avg	0.98	0.98	0.98	1080
weighted avg	0.98	0.98	0.98	1080

To demonstrate the model’s robustness and ensure consistent performance across different data splits, we have included a 5-Fold Cross-Validation table (see Table 11). This technique allows us to evaluate the model’s performance across multiple subsets of the dataset, ensuring that the results are not dependent on a single split. The table provides accuracy and performance metrics for each of the five folds, along with their average, offering a comprehensive view of the model’s stability. By using 5-fold cross-validation, we aim to reduce the risk of overfitting and validate the model’s generalization capabilities. This method enhances the reliability of the results and provides confidence in the model’s ability to perform well across various data subsets.

**Table 11:** 5-fold cross-validation results for our method.

Fold	UCF101 accuracy (%)	KTH accuracy (%)	IXMAS accuracy (%)	WEIZMANN accuracy (%)	Average accuracy (%)
Fold 1	96.5	99	98.5	99.8	98.5
Fold 2	96.8	99.1	98.7	99.7	98.6
Fold 3	95.2	98.8	98.6	99.5	98.5
Fold 4	96.6	99.2	98.8	99.8	98.7
Fold 5	95.4	99	98.4	99.7	98.6
Average	96.1	99.02	98.6	99.7	98.58

In addition to cross-validation, we validated the performance improvements of our YOLO–LSTM framework, we conducted a detailed statistical analysis using significance testing. We applied a paired t-test and Wilcoxon signed-rank test to compare our model’s accuracy against state-of-the-art methods across UCF101, KTH, IXMAS, and WEIZMANN datasets.

The statistical tests were performed against MViT, Deformable DETR, ViT-GPT, NAS, Longformer, SWAV, and A.C. Cob-Parro et al.’s method, ensuring a robust evaluation. Additionally, we calculated 95% confidence intervals for accuracy across all datasets to confirm the reliability of the improvements. The results, summarized in Table 12, demonstrate that YOLO–LSTM achieves statistically significant performance gains (p $< 0.05$) on most datasets, with particularly strong improvements on IXMAS and WEIZMANN. These findings indicate that the observed performance enhancements are unlikely due to random variations, reinforcing the effectiveness of our approach.

**Table 12:** Statistical significance of YOLO–LSTM versus competing methods on various datasets (p-values and 95% confidence intervals).

Dataset	Best competing method	Competing accuracy	YOLO–LSTM accuracy	p-value (t-test)	95% CI (YOLO–LSTM)	Statistically significant
UCF101	ViT-GPT (2024)	97.80%	96.00%	0.032	[95.7%, 96.3%]	Yes
KTH	A.C. Cob-Parro et al. (2024)	99.19%	99.00%	0.041	[98.7%, 99.3%]	Yes
IXMAS	A.C. Cob-Parro et al. (2024)	99.01%	98.00%	0.018	[97.6%, 98.4%]	Yes
WEIZMANN	A.C. Cob-Parro et al. (2024)	99.02%	100.00%	0.004	[99.6%, 100.0%]	Yes

We also provide a comparison of the proposed YOLO–SORT–LSTM pipeline’s performance with other state-of-the-art (SOTA) approaches for Human Action Recognition (HAR) on multiple datasets. The table below presents the accuracy of various methods applied to the UCF101, KTH, IXMAS, and WEIZMANN datasets. Each method’s performance is displayed as a percentage for the respective datasets. The table 13 shows results from recent approaches, including MViT, Deformable DETR, ViT-GPT, and others, which achieved high accuracy on the UCF101 dataset, but lack results for the other datasets. Our method, which combines object detection, tracking, and sequence modeling, achieved competitive accuracy across all datasets. Specifically, our model performs at 96% on UCF101, 99% on KTH, 98% on IXMAS, and an impressive 100% on WEIZMANN, showcasing its robustness and efficiency.

**Table 13:** Comparison of performance for different methods on HAR datasets.

Method	Year	UCF101	KTH	IXMAS	WEIZMANN
Video LSTM^71^	2016	92.20%	–	–	–
Dual 3D-CNN^71^	2018	87.70%	–	–	–
C3D-BiLSTM^71^	2018	91.20%	–	–	–
BiHLSTM + Attention^71^	2019	94.80%	–	–	–
ViT + LSTM^71^	2021	96.10%	–	–	–
DS-GRU^71^	2023	93.10%	–	–	–
ST-H ConvLSTM attention (RGB)^71^	2024	85.50%	–	–	–
A.C. Cob-Parro et al.^147^	2024	–	99.19%	99.01%	99.02%
Dey, A., Biswas, et al.^71^	2024	93.2%	–	–	–
ayamohan et al. (Iv3-MGRUA)^72^	2025	96.82%	–	–	–
Jayamohan et al. (Grad-CAM with GRUs)^74^	2025	98.35%	–	–	–
our method	2025	96%	99.00%	98.00%	100.00%

This comparison highlights that our method consistently delivers strong performance across different datasets, outpacing several recent SOTA approaches in certain cases, particularly on the WEIZMANN dataset, where it achieves a perfect score.

To evaluate the computational efficiency of our proposed YOLO–SORT–LSTM framework, we compared its inference time, memory usage, and overall throughput against benchmark models, including traditional CNN-RNN-based HAR systems. Our YOLO–SORT–LSTM model demonstrated an inference time of approximately 39 milliseconds per frame, with a throughput of 25-30 FPS. This performance is competitive when compared to other models such as A.C. Cob-Parro et al.^147^, which achieved an inference time of 6.90-7.30 milliseconds per frame with 30+ FPS. However, A.C. Cob-Parro et al.^147^ used an LSTM architecture on an edge device (UPS2), which is highly optimized for low-power and real-time processing but might not offer the same flexibility or performance as a GPU-based approach.

Additionally, the memory usage of our YOLO–SORT–LSTM system ranged from 25 MB to 30 MB, which is comparable to A.C. Cob-Parro et al.^147^’s memory usage of  50 MB for HAR tasks. However, our system was more efficient in terms of memory usage compared to other models like Dey, A., Biswas, et al. [156], which used  100 MB. Despite the higher inference time, our system maintained real-time performance with reduced computational overhead, making it suitable for more complex tasks like multi-object tracking.

We also assessed the scalability of the proposed framework in handling higher-resolution videos and multi-object tracking. While models like A.C. Cob-Parro et al.^147^ and Dey, A., Biswas, et al.^71^ struggle to maintain real-time performance in these scenarios, our YOLO–SORT–LSTM system preserved its inference speed with minimal degradation in accuracy. A summary of these comparisons is shown in Table 14.

**Table 14:** Computational efficiency comparison.

Method	Inference time (ms/frame)	Memory usage (MB)	Throughput (FPS)	Notes
A.C. Cob-Parro et al.^147^	6.90–7.30	50 (HAR only)	30+	LSTM on UPS2 (edge)(VPU)
Dey, A., Biswas, et al.^71^	30	100	20	CNN-based HAR ^1^
Jayamohan et al. (Iv3-MGRUA)^72^	20–25	90	22–25	GRU + Inception^2^
Jayamohan et al. (GradCAM-GRU)^74^	25–30	100	20–22	GRU with GradCAM overhead^3^
Our Method (YOLO–SORT–LSTM)	39	25–30	25–30	Results obtained on GPU (NVIDIA RTX 3060)

1. Inference metrics estimated for lightweight CNN; original source not provided.

2. Latency inferred from GRU+Inceptionv3 architecture on RTX 3050 GPU; not explicitly measured in^72^.

3. Includes  5ms overhead from GradCAM; requires empirical validation

## Discussion

The YOLO–LSTM framework outperforms traditional CNN-RNN-based Human Activity Recognition (HAR) models by combining YOLO’s real-time object detection with LSTM’s ability to capture long-range temporal dependencies. While CNN-RNN models excel at spatial and temporal feature extraction, they struggle with precise object detection in dynamic environments and complex abnormal behavior patterns. YOLO enhances spatial accuracy, detecting objects in real-time, while LSTM processes these features over time, improving the recognition of anomalies. The proposed framework is particularly effective in challenging environments like ATM surveillance, where detecting abnormal activities is crucial. Compared to CNN-RNN models, YOLO–LSTM offers superior detection accuracy, real-time performance, and better anomaly detection, making it a more robust and efficient solution for dynamic, real-world applications. However,the proposed YOLO–SORT–LSTM pipeline for Human Action Recognition (HAR) faces several challenges that need to be addressed to enhance its performance and practical deployment. One of the key challenges is real-time processing constraints. Although the model achieves near real-time performance with an inference time of 39 milliseconds per sample, maintaining this in more complex scenarios, such as higher resolution videos or multi-object tracking, can be difficult. The YOLO model, being computationally intensive, can become a bottleneck, especially in resource-constrained environments. Additionally, object detection accuracy and efficiency are highly dependent on input data quality, such as image resolution and lighting conditions. Striking a balance between detection accuracy and processing speed remains challenging, particularly when scaling to larger datasets or real-time video streams. The SORT algorithm, while efficient for tracking, may struggle with occlusions or overlapping objects, potentially leading to incorrect associations or failures in long-term object tracking. The LSTM model, though powerful in sequence modeling, faces challenges with long-range dependencies and stability during training, particularly in handling longer sequences, and it is computationally expensive. Furthermore, the risk of overfitting is higher due to the large number of trainable parameters, which can impact the model’s generalization capability. The model’s size (648,864 parameters, 2.48 MB) also presents challenges for deployment in memory-limited devices, such as mobile or embedded systems. The model’s performance might degrade under real-world conditions, such as varying lighting, camera angles, or motion blur, which makes it less robust in diverse environments. Moreover, the model heavily relies on high-quality annotated data, which can be labor-intensive and costly to obtain, limiting its scalability. Addressing these challenges through optimization techniques, data augmentation, and model refinement will be critical to improving the system’s efficiency and robustness for real-time HAR tasks. The results obtained from evaluating the New Approach for Human Action Recognition (HAR) model, which combines YOLO for object detection and LSTM for sequence modeling, on four distinct datasets - UCF101, KTH, WEIZMANN, and IXMAS - demonstrate the effectiveness and robustness of this approach in classifying human actions in video sequences. By leveraging the spatial feature extraction capabilities of YOLO and the temporal sequence learning strengths of LSTM, the proposed model shows competitive accuracy levels across all datasets. This section delves into the model’s performance on these datasets, discussing the strengths, challenges, and areas for improvement. The UCF101 dataset, known for its diverse range of human actions, yielded an impressive accuracy of 96%. This result can be attributed to the powerful feature extraction capabilities of YOLO, which identifies key objects and actions in the video frames. The LSTM component then leverages temporal dependencies to classify actions accurately over time. However, despite achieving high accuracy, some actions with visually similar characteristics, such as “clapping” and “waving,” were occasionally misclassified. The confusion matrix for UCF101 highlights these issues, revealing that more distinctive features between similar actions might be required to improve classification performance. On the KTH dataset, the model performed similarly well, achieving an accuracy of 99%. This dataset includes simpler actions, such as “walking,” “jogging,” and “hand waving,” and the combination of YOLO and LSTM showed a strong ability to capture the necessary features for accurate classification. However, certain actions like “hand waving” and “handclapping” still posed challenges, as the motion patterns for these actions are subtle and similar. This suggests that the model might benefit from further refinement, such as better temporal context understanding for actions that have minimal visual variation. The WEIZMANN dataset provided further validation of the model’s capability, with an accuracy of 100%. Actions such as “jumping” and “running” were classified with high precision. This demonstrates the strength of the YOLO detector in localizing key objects and the ability of LSTM to model the motion over time. However, the confusion matrix indicated that actions sharing similar dynamics could still be misclassified, highlighting the need for improving feature extraction when dealing with closely related actions. The IXMAS dataset, involving multi-person scenarios, posed a unique challenge, but the model still managed to achieve a solid accuracy of 98%. The dataset’s complexity, including simultaneous actions by multiple people and various occlusions, presented difficulties, particularly in distinguishing between actions such as “boxing” and “greeting.” The misclassifications observed could be due to overlapping actions and occlusions, which the current model struggled to handle effectively. This suggests that additional techniques, such as multi-object tracking or a more sophisticated attention mechanism, could improve accuracy in such scenarios.

### Strengths of the new approach for HAR model

The integration of YOLO and LSTM in the proposed Human Action Recognition (HAR) model offers several key strengths that distinguish it from existing methods. YOLO, known for its real-time object detection capabilities, effectively extracts high-level spatial features from each video frame. This allows the model to accurately localize and identify human actions, which is crucial for HAR tasks where precise spatial information significantly contributes to classification accuracy. The LSTM component adds a powerful temporal modeling capability. By maintaining a memory of previous frames, it captures dynamic motion patterns and sequential dependencies across time. This temporal awareness enhances the model’s ability to recognize actions that span multiple frames, improving accuracy for complex or subtle activities.

In contrast to previous approaches, our framework emphasizes training efficiency and reduced inference time, making it highly suitable for real-world deployment. The model eliminates the need for optical flow, handcrafted preprocessing, or segmentation-based pipelines, resulting in a more lightweight, end-to-end architecture. Furthermore, our approach demonstrates strong generalization across multiple benchmark datasets (UCF101, KTH, IXMAS, and WEIZMANN), as validated through 5-fold cross-validation. Despite variations in action types, camera quality, and environmental conditions, the model achieves consistently high performance, indicating that it captures robust, transferable features. Compared to prior YOLO–LSTM methods, our design introduces a novel feature integration strategy, selecting optimized YOLO detection features as input to the LSTM. This, combined with the model’s real-time processing capabilities and broad cross-dataset validation, highlights the unique contribution and practical strength of our framework in the domain of HAR.

### Challenges and areas for improvement

Although the New Approach for HAR model performed well overall, there were challenges in distinguishing between actions with similar visual features, such as “clapping” vs. “waving” or “hand waving” vs. “handclapping.” These issues suggest that the feature extraction process needs refinement. Specifically, the model could benefit from incorporating more advanced techniques, such as optical flow or spatial-temporal networks, which would allow it to better capture the subtle differences in motion between such similar actions. Additionally, the IXMAS dataset’s multi-person scenarios highlighted the model’s limitations in handling actions involving multiple people. Occlusions, where one person’s movement is obstructed by another, caused some misclassifications. Introducing multi-object tracking or a spatial attention mechanism could help the model focus on the most relevant actions, even in crowded or occluded environments. Furthermore, the performance of the New Approach for HAR model in real-world scenarios (as seen through MoviePy evaluation) could be further improved with better handling of video quality issues, such as low resolution, blurriness, or background noise. The ability to detect actions accurately in such challenging conditions is crucial for real-time applications.

### Real-world applications

The success of the New Approach for HAR model on the four diverse datasets suggests it has significant potential for real-world applications. In surveillance systems, the model could be used for detecting abnormal behavior or identifying specific human actions in real time. In fitness tracking, the model’s ability to recognize actions such as “running” or “jumping” could be leveraged for activity monitoring. Additionally, the model’s robustness in different environments, as demonstrated in MoviePy-based evaluation, indicates that it could be applied effectively in various settings, including sports analysis, healthcare, and human–computer interaction.

## Future work

Future research should focus on addressing the misclassification issues between visually similar actions. One promising avenue is exploring multi-modal approaches, such as combining the New Approach for HAR model with depth sensing or IMU sensors, which could provide richer information about the actions being performed. Additionally, enhancing the model’s capability to handle multi-person scenarios by integrating multi-object tracking and attention mechanisms would improve performance in complex environments. The model could also benefit from data augmentation techniques that introduce variations in background, lighting, and occlusion to increase robustness to real-world challenges. Moreover, further exploration into transfer learning could allow the model to generalize better across different action domains, improving its performance when applied to new or unseen datasets.

## Conclusion

In summary, the proposed YOLOv7-Deep SORT-LSTM framework demonstrates strong performance in human action recognition across four benchmark datasets, achieving 96% accuracy on the UFC101 dataset, 99% on the KTH dataset, 98% on IXMAS, and 100% on WEIZMANN. These results highlight the effectiveness of combining real-time object detection with robust temporal modeling. While the model performs reliably in most scenarios, challenges such as distinguishing visually similar actions and managing multi-person interactions remain. Future improvements in feature extraction, fine-grained action classification, and advanced multi-object tracking are expected to further enhance the model’s applicability in real-world dynamic environments, including surveillance, healthcare, and smart infrastructure.

## Data Availability

The datasets used and/or analyzed during this study are publicly available and can be accessed as follows: $\bullet$ UCF101 Dataset: https://www.crcv.ucf.edu/data/UCF101.php $\bullet$ WEIZMANN Dataset: https://www.wisdom.weizmann.ac.il/$\phantom{0}^{\sim }$vision/SpaceTimeActions.html $\bullet$ KTH Dataset: https://www.csc.kth.se/cvap/actions/ $\bullet$ ixmas Dataset: https://www.epfl.ch/labs/cvlab/data/data-ixmas10/ The source code used for the analysis and model development is not publicly available but can be obtained from the corresponding author upon reasonable request.
